# Real‐Time Tracking of ASCT2‐Mediated Glutamine Uptake in Living Tumors With a Bioorthogonal Bioluminescent Probe

**DOI:** 10.1002/advs.202507057

**Published:** 2025-08-10

**Authors:** Bing‐Jun Zhou, Ji‐Lei Zhao, Fan Yin, Wen‐Da Chen, Yi Sun, Ying‐Ying Ren, Yi‐Min Chen, Tian Xie, Chong Duan, Jian‐Liang Zhou

**Affiliations:** ^1^ School of Pharmacy Hangzhou Normal University Hangzhou China; ^2^ Zhejiang Provincial Key Laboratory of Anti‐Cancer Chinese Medicines and Natural Medicines Hangzhou Normal University Hangzhou China; ^3^ Innovation Research Institute of Traditional Chinese Medicine Shanghai University of Traditional Chinese Medicine Shanghai China

**Keywords:** ASCT2, bioluminescent probe, glutamine uptake, real‐time monitoring

## Abstract

Glutamine addiction, as a hallmark of tumor metabolism, drives malignant progression via proliferation, survival, and metastasis. Alanine‐serine‐cysteine transporter 2 (ASCT2), the primary glutamine transporter, is overexpressed in tumors to meet metabolic demands, making it a promising therapeutic target. Accurately monitoring ASCT2‐mediated glutamine uptake is essential for investigating tumor metabolism and developing ASCT2‐targeted therapeutics. However, current methods lack specificity, require laborious sample processing, and do not support real‐time measurements in living systems. To overcome these issues, BLGLN is designed, an innovative bioluminescent reporter system exploiting Staudinger ligation. BLGLN comprises two components: 1) BL568, a caged D‐luciferin derivative protected with 2‐diphenylphosphinobenzoic acid, and 2) AA201, an azide‐modified glutamine mimetic taken up by ASCT2. Once inside, AA201 undergoes Staudinger ligation with membrane‐permeable BL568, releasing D‐luciferin that is converted by luciferase into a bioluminescent signal, allowing real‐time tracking of ASCT2‐dependent glutamine uptake in tumors. BLGLN provides simplified synthesis, eliminates complex sample preparation, and enables real‐time tracking and evaluation of glutamine uptake rate in living tumors.

## Graphical abstract

A bioluminescent probe, BLGLN, comprising BL568 and ASCT2‐uptaken AA201, enables real‐time monitoring and evaluation of ASCT2‐mediated glutamine uptake rates in living tumors via Staudinger ligation, aiding tumor metabolism studies and targeted therapeutics.

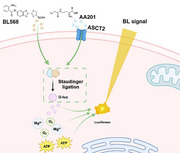

## 1 Introduction

Tumor metabolic reprogramming refers to the active modifications in metabolic pathways and flux undertaken by cancer cells to facilitate their initiation, progression, invasion, metastasis, and resistance to therapy. A defining characteristic of this reprogramming is the substantial uptake of glutamine by tumor cells, which plays essential roles in biosynthesis, energy provision, maintenance of redox homeostasis, and regulation of signaling pathways.^[^ ^1^ ^]^ To meet their heightened demand for glutamine, numerous tumor types upregulate the expression of the alanine‐serine‐cysteine transporter 2 (ASCT2), encoded by SLC1A5 gene, which serves as the primary transporter for glutamine uptake^[^ ^2^, ^3^, ^4^, ^5^, ^6^, ^7^ ^]^ This overexpression is strongly associated with key tumor attributes, including survival,^[^ ^2^, ^8^, ^9^ ^]^ progression,^[^ ^2^, ^3^, ^4^, ^9^ ^]^ drug resistance,^[^ ^10^ ^]^ and poor prognosis.^[^ ^2^, ^11^ ^]^ Inhibition of ASCT2 markedly reduces intracellular glutamine levels, suppresses tumor progression,^[^ ^3^, ^4^, ^9^, ^12^ ^]^ and enhances the antitumor efficacy of other therapeutic agents.^[^ ^10^, ^13^, ^14^ ^]^ Consequently, ASCT2 has emerged as a promising therapeutic target in oncology,^[^ ^15^, ^16^, ^17^, ^18^, ^19^ ^]^ sparking significant interest in developing strategies to block ASCT2‐mediated glutamine uptake and establishing methods to monitor and evaluate ASCT2‐dependent uptake, underscoring the transporter's potential as a focal point for advancing cancer treatment.

Traditional analytical techniques, such as liquid chromatography‐mass spectrometry (LC‐MS),^[^ ^20^, ^21^ ^]^ glutamine assay kit,^[^ ^22^ ^]^ and radiolabeled glutamine uptake assays (^3^H/^14^C),^[^ ^23^, ^24^ ^]^ offer quantitative assessments of intracellular glutamine levels. However, these methods are limited by two significant drawbacks: they require disruptive sample preparation procedures, including cell lysis and metabolite extraction, and they are incapable of monitoring glutamine uptake in both in vitro and in vivo settings. Positron emission tomography (PET) probes are currently the primary tools for in vivo monitoring and quantification of glutamine uptake. These probes primarily utilize ^11^C‐labeled glutamine or ^18^F‐substituted glutamine derivatives. Despite their excellent tissue penetration capabilities, PET probes possess notable limitations. For instance, ^11^C‐labeled L‐5‐[^11^C]Gln^[^ ^25^ ^]^ exhibits a short half‐life of ≈20 min, whereas ^18^F‐substituted probes, such as [^18^F]‐(2S,4R)‐4‐FGln,^[^ ^26^ ^]^ [18F]‐4‐(3‐fluoropropyl)glutamine,^[^ ^27^ ^]^ and (2S,4S)‐4‐[^18^F]FEBGln,^[^ ^28^ ^]^ overcome the half‐life constraint but encounter difficulties with low radiolabeling efficiency.^[^ ^28^, ^29^ ^]^ Moreover, PET probes cannot enable real‐time monitoring of cellular glutamine uptake in vitro since they are not “turn‐on” probes and require cell lysis and complex sample preparation. ^[^ ^28^, ^30^ ^]^ Additionally, owing to the gamma radiation they emit, their application requires specialized facilities and trained personnel. Another critical consideration is that, for certain PET probes, incorporating lipophilic groups into the amino acid side chains shifts their cellular uptake preference from ASCT2 (the primary glutamine transporter) to LAT1, potentially compromising target specificity.^[^ ^28^ ^]^

**Figure 1:**
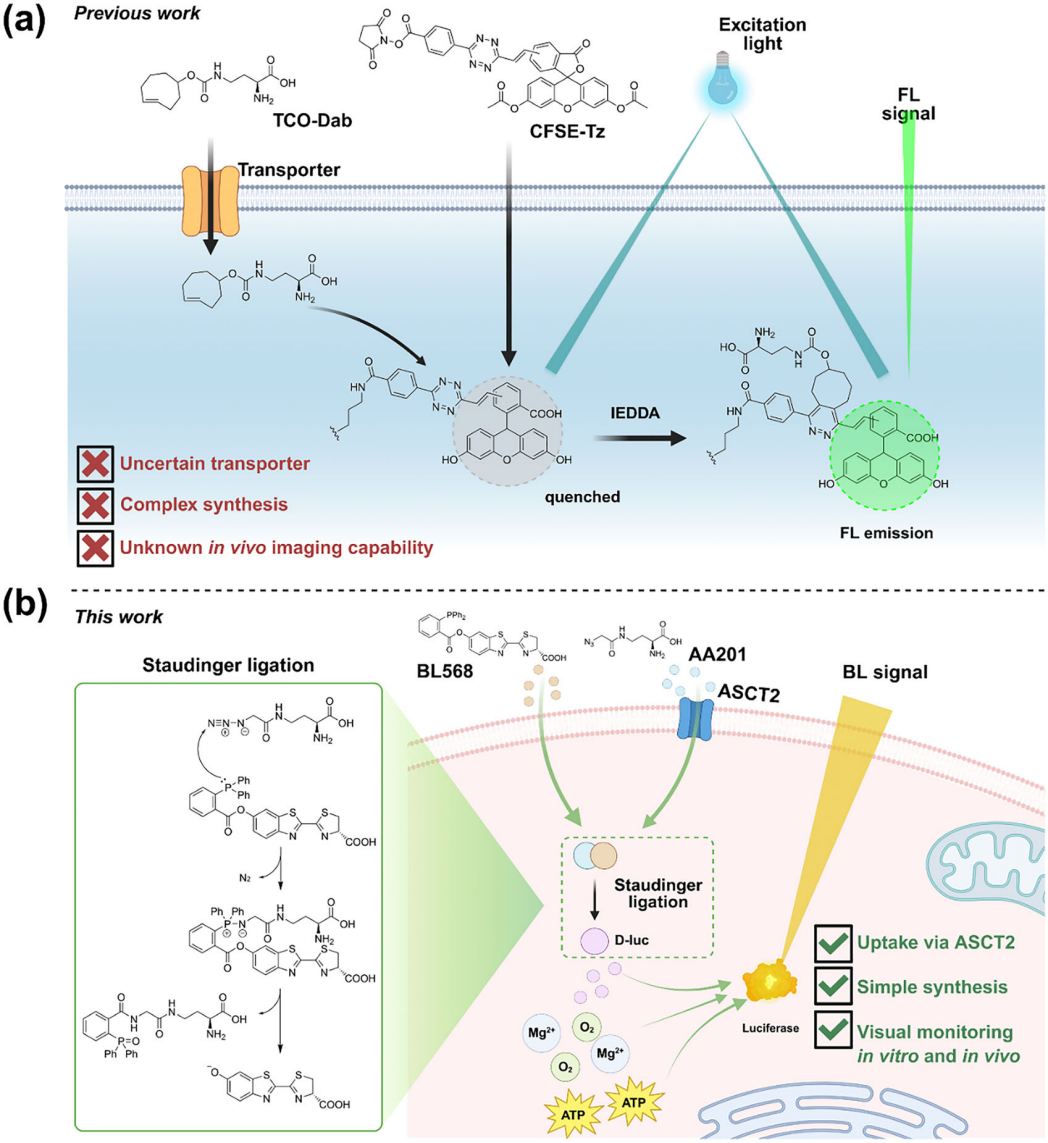
The probes to monitor cellular glutamine uptake. a) Previous work reported by Wang et al.^[^ ^31^ ^]^ b) This work.

Recently, Wang et al.^[^ ^31^ ^]^ introduced CFSE‐Tz, a fluorescent probe designed for the visual monitoring of amino acid uptake in live cells, leveraging the inverse electron‐demand Diels‐Alder (IEDDA) reaction (**Figure** **1**a). This probe employs carboxyfluorescein diacetate succinimidyl ester (CFSE) conjugated with a tetrazine (Tz) moiety, which effectively quenches CFSE fluorescence. Subsequently, a trans‐cyclooctene‐functionalized amino acid, TCO‐Dab, was synthesized to undergo IEDDA with CFSE‐Tz, thereby liberating CFSE and restoring fluorescence. In comparison to PET probes, this fluorescent methodology allows for direct, in vitro visualization of cellular amino acid uptake and has been successfully applied to single‐cell imaging. However, the absence of conclusive evidence supporting ASCT2 mediation of TCO‐Dab transport raises concerns regarding the reliability of this approach for specifically tracking ASCT2‐mediated glutamine uptake. Furthermore, the feasibility of in vivo visualization using this probe remains to be established.

Current methodologies lack probes capable of specifically monitoring ASCT2‐mediated glutamine uptake dynamics while permitting evaluation of glutamine uptake rate in both in vitro and in vivo systems. To address this, we developed a biorthogonal bioluminescent probe, BLGLN, for real‐time monitoring and evaluation of glutamine uptake (Figure 1b). BLGLN is designed based on Staudinger ligation, utilizing an azido‐functionalized amino acid, AA201, which exhibits structural similarity to glutamine and features easy synthesis and purification. ASCT2 inhibition and knockdown experiments confirmed AA201 transport via ASCT2, validating BLGLN as a reliable probe for ASCT2‐mediated glutamine uptake. These findings enabled the development of a BLGLN‐based method for real‐time monitoring and evaluating glutamine uptake, demonstrating its effectiveness, reliability, and rapid response (maximum signal intensity achieved within 14 min) in tumor cells. Furthermore, in vivo imaging confirmed that BLGLN enables tumor visualization and evaluates glutamine uptake through bioluminescence intensity. BLGLN offers a novel approach for real‐time monitoring of tumor glutamine uptake and evaluating tumor glutamine uptake rate both in vitro and in vivo, characterized by simplified synthesis and preparation, elimination of complex sample preparation, and the capability for real‐time, high‐throughput monitoring of glutamine uptake in living tumors.

## 2 Result and Discussion

### 2.1 Probe Design

To monitor ASCT2‐mediated glutamine uptake, the probe must be designed to retain structural similarity to native glutamine, ensuring efficient transport via ASCT2. For bioorthogonal probes based on the IEDDA reaction, the introduction of tetrazine or trans‐cyclooctene is necessary.^[^ ^32^ ^]^ However, the bulky volume and strong lipophilicity of these groups are unfavorable for maintaining the structural similarity between the probe and native glutamine. In contrast, the Staudinger ligation utilizes a triphenylphosphine‐azide reaction^[^ ^32^ ^]^ where the compact azide group minimizes structural perturbation, maintaining close resemblance between the glutamine derivative and native glutamine. Therefore, we selected the Staudinger ligation as the trigger mechanism for the probe, utilizing an azide‐modified glutamine along with a 2‐diphenylphosphinobenzoic acid‐protected reporter molecule.

The D‐luciferin/luciferase bioluminescence method is extensively employed for both in vivo and in vitro visualization imaging.^[^ ^33^ ^]^ Compared to fluorescence and chemiluminescence methods, the bioluminescence method offers high sensitivity,^[^ ^34^ ^]^ high signal specificity,^[^ ^35^ ^]^ and cost‐effectiveness.^[^ ^36^ ^]^ Consequently, BLGLN was designed utilizing the D‐luciferin/luciferase bioluminescence method. BLGLN is composed of two key components (Figure 1b): 1) a 2‐diphenylphosphinobenzoic acid‐protected D‐luciferin derivative (BL568), designed for cellular entry via passive diffusion,^[^ ^37^ ^]^ and 2) an azido‐functionalized glutamine derivative, engineered for ASCT2‐mediated transport. Upon intracellular co‐localization, these components undergo a Staudinger ligation, releasing D‐luciferin, the substrate for luciferase. Subsequent luciferase‐catalyzed oxidation of D‐luciferin generates a quantifiable bioluminescent signal, enabling real‐time monitoring of ASCT2‐mediated glutamine uptake in tumor cells.

**Figure 2:**
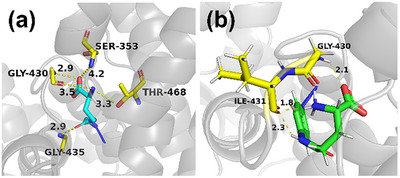
Molecular docking of ASCT2 (PDB: 6GCT) with a) AA201 (blue molecule) and b)AA263 (green molecule). Amino acid residues are represented by the color yellow. The hydrogen bonds are represented by yellow dotted lines.

A critical consideration is the capacity of glutamine‐derived azido compounds to bind and be transported by ASCT2, essential for cellular uptake and subsequent biological applications. To address this, AA201 was rationally designed to emulate glutamine's binding mode with ASCT2, preserving crucial interactions for transporter recognition and substrate translocation. Molecular docking simulations confirmed that AA201 recapitulates key glutamine binding interactions (**Figure** **2**a): 1) carboxyl group hydrogen bonding with Gly430‐NH and Ser353‐OH; 2) amino group dual hydrogen bonding with Gly430‐CO and Thr468‐OH; and 3) amide carbonyl hydrogen bond acceptance from Gly435‐NH. This close mimicry of the native substrate's binding mode strongly indicates AA201's retention of structural determinants necessary for ASCT2‐mediated transport. Furthermore, leveraging the ASCT2 inhibitor GPNA, we synthesized AA263 by strategically substituting its nitro group with an azido moiety. Molecular docking simulations revealed a distinct binding mode for AA263 (Figure 2b): 1) carboxyl group hydrogen bonding with Gly430‐NH, and 2) amino and amide group hydrogen bonding with Ile431‐CO. While exhibiting a binding mode distinct from glutamine, AA263's preserved hydrogen bonding with Gly430 and Ile431 suggests binding affinity and potential ASCT2‐mediated transport.

### 2.2 Synthesis of Probes

**Figure 3:**
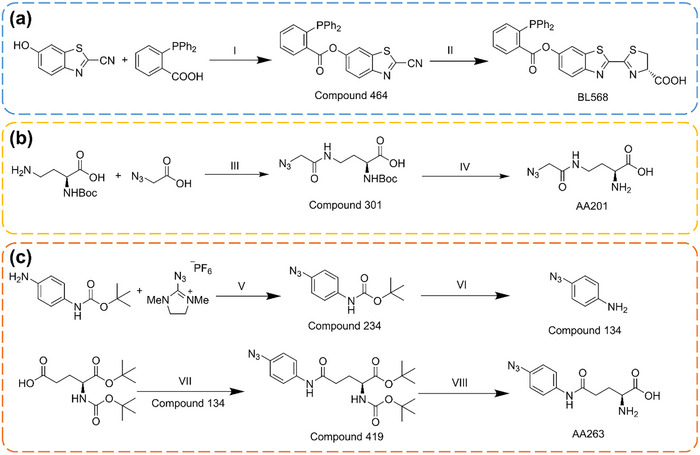
Synthesis pathway of a) BL568, b) AA201, and c) AA263. I: EDCI, DMAP, DCM, rt. II: D‐Cys, K_2_CO_3_, DCM, H_2_O, MeOH, rt. III: TSTU, DIPEA, H_2_O, ACN, rt. IV: 4M HCl/ 1,2‐Dioxane, DCM, rt. V: DMAP, ACN, 30 °C. VI: 4M HCl/ 1,2‐Dioxane, MeOH, rt. VII: EDCI, DMAP, DMSO, rt. VIII: 4M HCl/ 1,2‐Dioxane, DCM, rt.

BL568 was synthesized according to a previously established protocol (**Figure** **3**a).^[^ ^38^ ^]^ The synthetic route involved: 1) an ester condensation between 6‐hydroxy‐2‐cyanobenzothiazole and diphenylphosphinobenzoic acid, yielding intermediate 464; and 2) subsequent condensation of 464 with D‐cysteine, with purification of the resulting product, BL568, via reverse‐phase high‐performance liquid chromatography (RP‐HPLC). The structure of BL568 was confirmed by ^1^H‐NMR (Figure S1a, Supporting Information), ^13^C‐NMR (Figure S1b, Supporting Information), ^31^P‐NMR (Figure S1c, Supporting Information), and high‐resolution mass spectrometry (HRMS, Figure S1d, Supporting Information), with spectral data consistent with the target compound.^[^ ^38^ ^]^

AA201 was synthesized via a two‐step reaction pathway (Figure 3b): 1) amide condensation between azidoacetic acid and Boc‐L‐2,4‐diaminobutyric acid, followed by direct deprotection using HCl/1,4‐dioxane without intermediate purification; and 2) purification of the resulting product, AA201, by RP‐HPLC. The structure of AA201 was confirmed by ^1^H‐NMR (Figure S2a, Supporting Information), ^13^C‐NMR (Figure S2b, Supporting Information), and HRMS (Figure S2c, Supporting Information), confirming its identity as the target compound. Notably, AA201 benefits from a streamlined synthetic procedure and facile preparation.

The synthesis of AA263 was accomplished via a four‐step reaction sequence (Figure 3c). In the first step, 2‐azido‐1,3‐dimethylimidazolinium hexafluorophosphate was employed to azidate the amino group of 4‐(tert‐butoxycarbonylamino)aniline, affording compound 234. Its structure was confirmed by ^1^H‐NMR (Figure S3a, Supporting Information). Subsequent removal of the Boc protecting group yielded compound 134, which was used directly in the next step without further purification. Next, amide condensation between compound 134 and Boc‐L‐glutamic acid 1‐tert‐butyl ester efficiently produced compound 419. Its structure was confirmed by ^1^H‐NMR (Figure S3b, Supporting Information). Finally, deprotection of compound 419 directly afforded the target molecule AA263, obviating the need for further purification. The structure of AA263 was confirmed by ^1^H‐NMR (Figure S3c, Supporting Information). However, experimental observations revealed that AA263 exhibited poor aqueous solubility and insufficient chemical stability, being prone to degradation. Consequently, AA263 was excluded from further development.

### 2.3 Development of BLGLN‐Based Glutamine Uptake Monitoring Method in Tumor Cells

**Figure 4:**
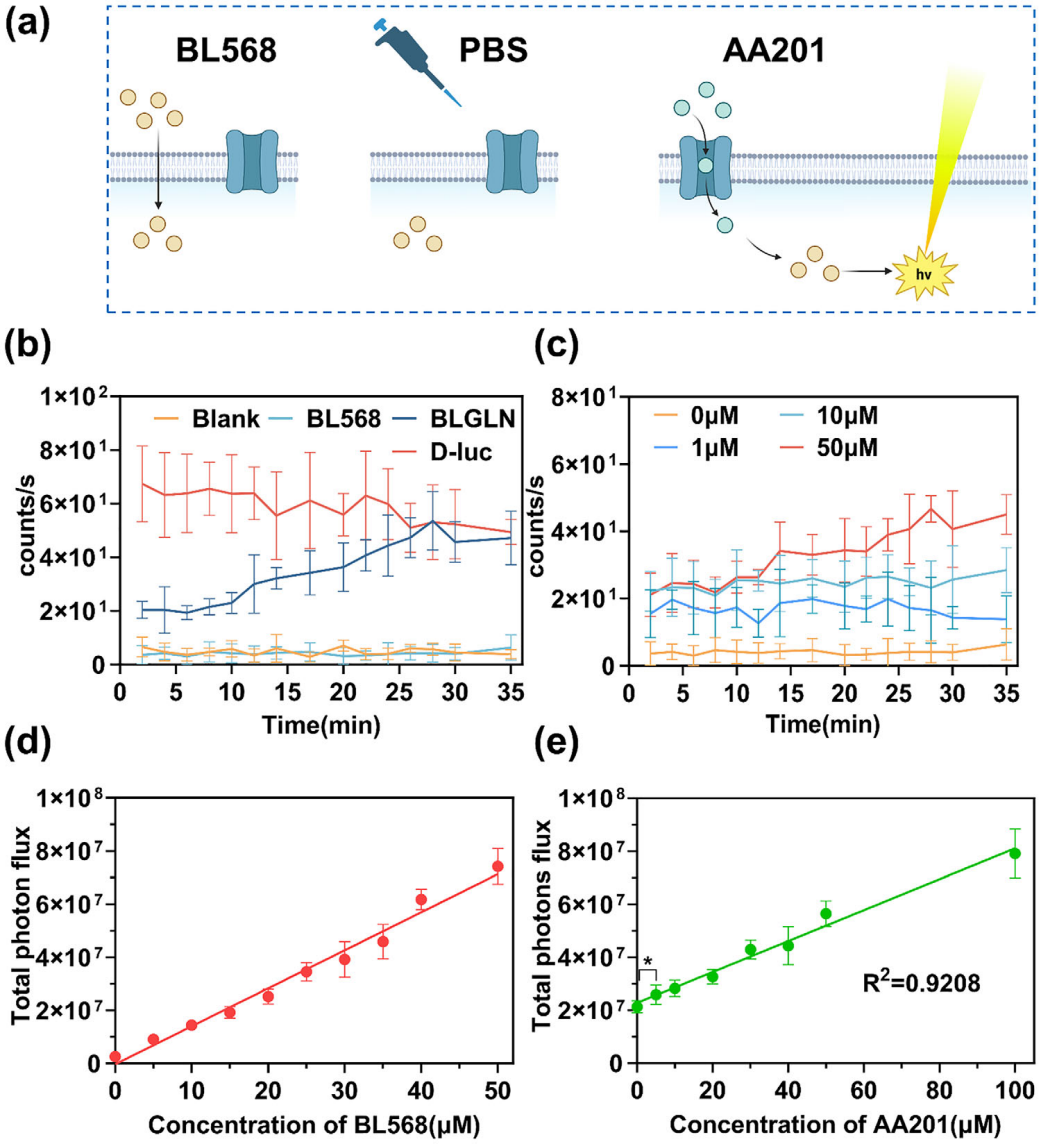
Verifying BLGLN excitation and optimizing BLGLN concentration. a) Steps for probe use. b) Bioluminescence verification. *n* = 6. c) BL Intensity of BLGLN at different concentrations of AA201. *n* = 6. d) Total photon flux at different concentrations of BL568. *n* = 6. e) Total photon flux of 14 to 30 min at different concentrations of AA201. *n* = 6. *p < 0.05.

Following probe synthesis, we developed a BLGLN‐based platform for monitoring glutamine uptake in tumor cells and optimized the experimental parameters (**Figure** **4**a). Initially, cytotoxicity assays were conducted to assess the safety profile of the probe components. Results demonstrated that BL568 exhibited no significant cytotoxicity toward colorectal cancer HCT116 cells at concentrations up to 50 µM, while AA201 displayed favorable biocompatibility at 100 µM (Figure S4, Supporting Information). Subsequently, a stable luciferase‐expressing HCT116 cell line was generated via lentiviral transfection, and its bioluminescent functionality was confirmed using D‐luciferin (Figure S5, Supporting Information).

Bioluminescent signal validation experiments revealed negligible signal detection in the BL568‐only and blank control groups, while the D‐luciferin group exhibited consistently high signal intensity. Notably, the AA201‐treated experimental group displayed a time‐dependent increase in bioluminescent signal, ultimately reaching levels comparable to the D‐luciferin group, validating the functionality of BLGLN (Figure 4b). Furthermore, concentration gradient experiments demonstrated a direct correlation between AA201 concentration and bioluminescent signal intensity (Figure 4c), establishing a basis for the development of an in vitro method for visualizing glutamine uptake.

To establish optimal experimental parameters, we systematically optimized BL568 and AA201 concentrations. Initially, with AA201 fixed at 50 µM, we assessed the bioluminescent response across varying BL568 concentrations. Bioluminescence (BL) intensity time course plot revealed signal stabilization after ≈14 min, with post‐stabilization signal intensity increasing proportionally to BL568 concentration (Figure S6a, Supporting Information). Total photon flux analysis corroborated this trend (Figure 4d). Based on a comprehensive evaluation of both cytotoxicity and signal intensity profiles, we identified 50 µM as the optimal working concentration for BL568. Using this standardized concentration, we then systematically optimized the AA201 concentration. Real‐time monitoring data demonstrated a similar time‐dependent stabilization of the bioluminescent signal, with intensity increasing alongside AA201 concentration (Figure S6b, Supporting Information). Total photon flux statistical analysis showed a strong linear correlation between signal intensity and AA201 concentration (R^2^ = 0.9208) (Figure 4e). Balancing cytotoxicity and signal response, 100 µM was determined as the optimal working concentration for AA201. In addition, at a low concentration of AA201 (5 µM), total photon flux showed a signal enhancement (*p *< 0.05, Figure 4e). These results demonstrate that BLGLN exhibits excellent detection sensitivity and shows high potential for biological applications.

**Figure 5:**
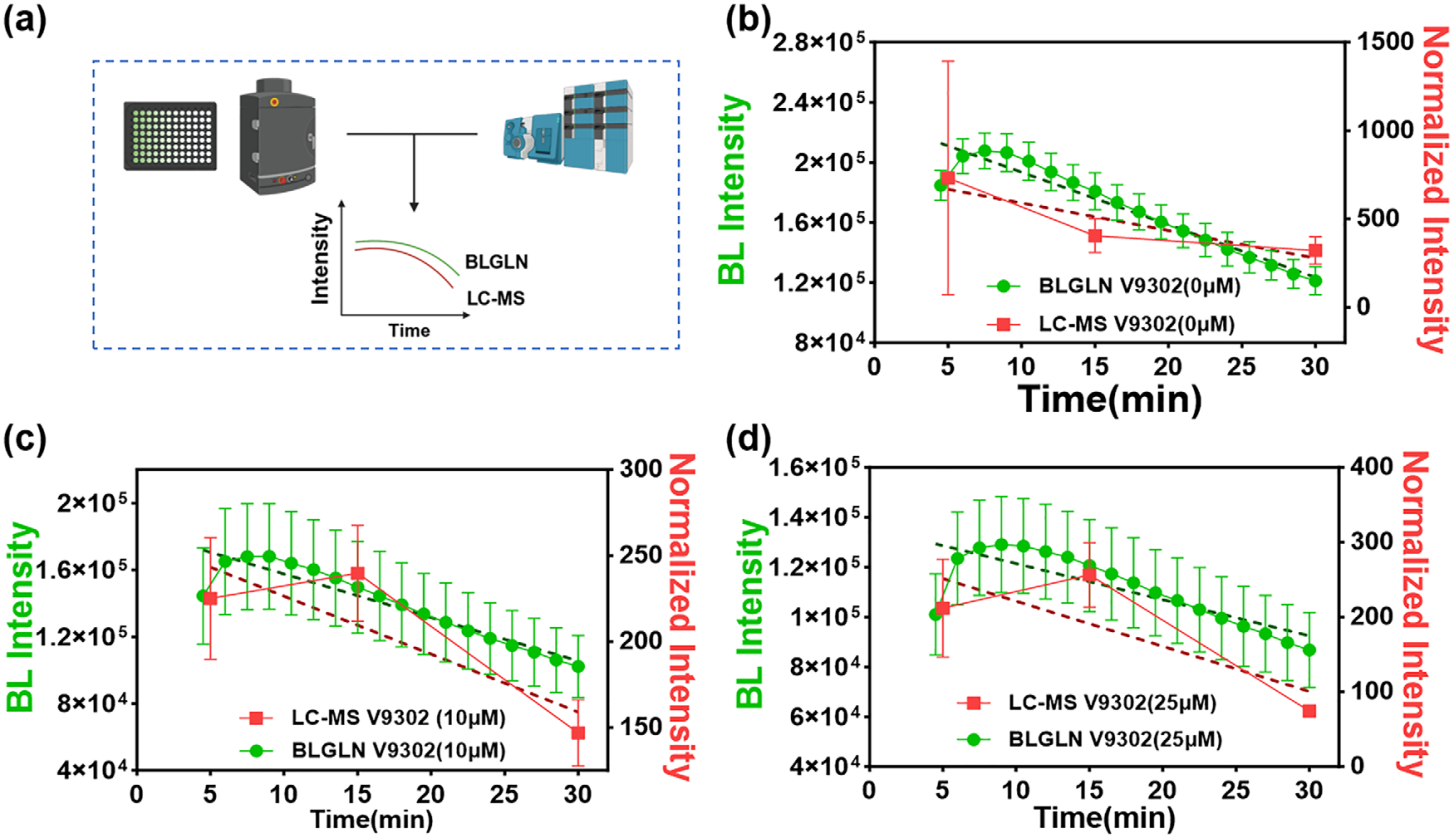
The BLGLN compared with LC‐MS. a) Detecting the result of ASCT2 transport capacity changing by BLGLN and isotopic labeling mass spectrometry. Changes in intracellular AA201 concentrations (measured by BLGLN) and intracellular isotope‐labeled glutamine levels (quantified via LC‐MS) following treatment with b) 0 µm V9302, c) 10 µm V9302, and d) 25 µm V9302 over the incubation period; green: BLGLN, *n* = 6; red: LC‐MS, *n* = 3.

To validate the detection reliability of BLGLN, a comparative analysis was performed against a conventional liquid chromatography‐mass spectrometry (LC‐MS) method (**Figure** **5**a). We first established a quantitative ultra‐performance liquid chromatography‐triple quadrupole mass spectrometry (UPLC‐QQQ‐MS) method to specifically detect [^13^C_5_, ^15^N_2_]‐L‐glutamine in tumor cells. Method validation demonstrated good linearity within the 10–1000 ng mL^−1^ concentration range (Figure S7, Supporting Information), with intra‐day (RSD = 3.75%) and inter‐day (RSD = 6.97%) precision meeting quantification standards. Subsequently, the BLGLN and LC‐MS methods were compared for their ability to evaluate the intracellular glutamine content trends in different concentrations of V9302, a selective ASCT‐2 inhibitor. In inhibitor‐treated cells, both the BL intensity and LC‐MS‐measured [^13^C_5_, ^15^N_2_]‐L‐glutamine levels exhibited consistent increasing and decreasing trends (Figure 5c,d). However, in untreated cells, partial discrepancies appeared between the two methods' trends (Figure 5b), likely reflecting distinct uptake and clearance pathways for AA201 versus glutamine. Notably, the overall BL intensity trend remained closely aligned with intracellular [^13^C_5_, ^15^N_2_]‐L‐glutamine content changes, demonstrating that BLGLN detection achieves comparable results to the conventional LC‐MS method.

To comprehensively characterize BL568's selectivity, we first performed fluorescence spectroscopy analysis, which demonstrated negligible signal responses to nitric oxide, cysteine, and other biologically relevant interferents (Figure S8, Supporting Information). Building on previous reports of esterase‐mediated probe activation,^[^ ^39^ ^]^ we further investigated potential background signals using class‐specific esterase inhibitors: NaF (carboxyesterases), PMSF (serine esterases), and DTNB (aryl esterases). Cellular experiments revealed that only DTNB treatment caused a significant reduction in background signal (Figure S9, Supporting Information), indicating that aryl esterase activity contributes to BL568's nonspecific signal generation. To eliminate this background interference, we introduced a normalized uptake rate calculation approach (the detailed formula is provided in Section 4.11).

Furthermore, extracellular BL568 could potentially interfere with BLGLN detection accuracy. To rigorously assess whether residual BL568 remained after PBS washing, we implemented a stringent validation protocol: after BL568 incubation and two PBS washes, cells were briefly (1 min) incubated with 100 µL KRH buffer, which was subsequently collected for HPLC analysis of extracellular BL568 content. The reference solution containing 10 µm BL568 exhibited a distinct UV absorption peak at 48.8 min, whereas blank KRH buffer showed no detectable signal at this retention time (Figure S10a, Supporting Information). Notably, the KRH buffer collected from washed cells also displayed no discernible peak at 48.8 min (Figure S10a, Supporting Information). To rigorously confirm complete removal of extracellular BL568, we quantitatively compared bioluminescence signals between double‐wash and triple‐wash protocols. Total photon flux analysis revealed no significant difference between the two conditions (Figure S10b, Supporting Information), demonstrating that two washes were sufficient to eliminate residual extracellular BL568 interference.

### 2.4 Confirmation of ASCT2 as the Primary Transporter of AA201 in Tumor Cells

**Figure 6:**
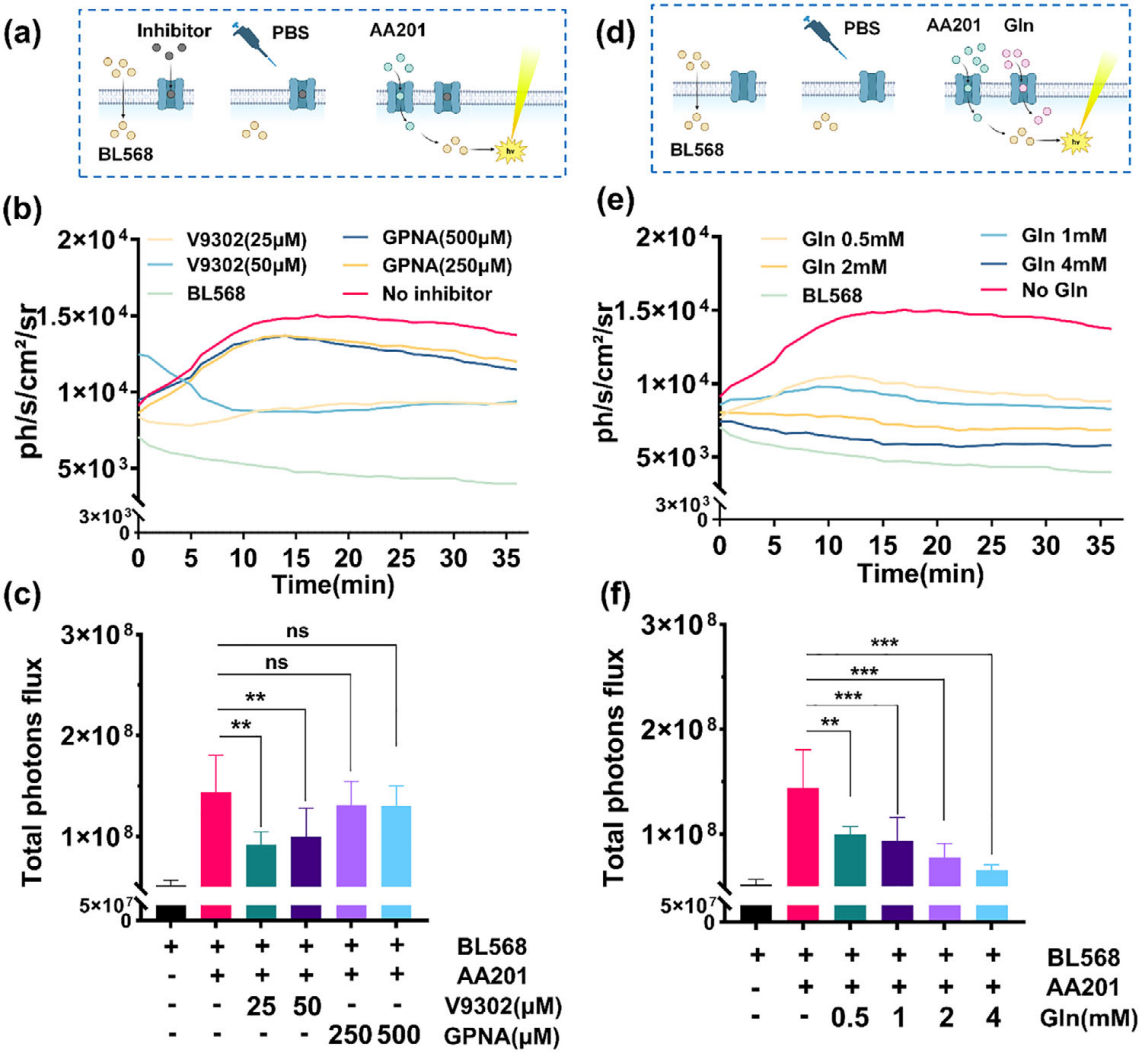
ASCT2 transporting capacity detected by BLGLN. a) Scheme for validating the detection of ASCT2 transport capacity by BLGLN using ASCT2 Inhibitors. b) BL intensity time course in cells pre‐treated with different inhibitors. c) Total photon flux in cells pre‐treated with different inhibitors. *n* = 6. d) Scheme for validating the detection of ASCT2 transport capacity by BLGLN using natural substrate. *n* = 6. e) BL intensity time course in cells treated with AA201 solution containing different concentrations of Gln. *n* = 6. f) Total photon flux in cells treated with AA201 solution containing different concentrations of Gln. *n* = 6. ns *p *> 0.05, ** *p *< 0.01, *** *p *< 0.001.

To determine whether ASCT2 constitutes the primary transporter responsible for cellular uptake of AA201, we conducted a systematic evaluation using ASCT2 inhibitors (V9302 and GPNA) and the natural substrate glutamine. The experimental design is outlined in **Figure** **6a**: cells were co‐incubated with BL568 and inhibitors, washed, and subsequently treated with 100 µM AA201 solution. Bioluminescent signal monitoring was performed using an in vivo imaging system. Inhibitor experiments revealed negligible signal in the BL568‐only group, while the BLGLN group exhibited significant bioluminescence (Figure 6b). Notably, V9302 treatment (V9302(25 µM) and V9302(50 µM)) markedly suppressed bioluminescent intensity, whereas GPNA treatment (GPNA (250 µM) and GPNA (500 µM)) showed weaker inhibition (Figure 6b). Total photon flux analysis corroborated these findings: V9302‐treated groups exhibited significantly lower total photon flux compared to the BLGLN group, while GPNA‐treated groups showed no significant difference (Figure 6c). The uptake rates demonstrated concordance with total photon flux (Figure S11a, Supporting Information). This observation aligns with the known potency differences between ASCT2 inhibitors, with the second‐generation V9302 demonstrating greater inhibitory efficacy than the first‐generation GPNA, confirming the probe's ability to accurately reflect inhibitor effects. To further validate the probe's detection function, a glutamine competitive inhibition experiment was performed (Figure 6d). Results showed a dose‐dependent decrease in bioluminescent intensity with increasing glutamine concentration (Figure 6e). Total photon flux analysis confirmed this trend: all glutamine‐containing experimental groups exhibited significantly reduced total photon flux compared to the BLGLN group, with greater reduction at higher glutamine concentrations (Figure 6f). The uptake rates demonstrated concordance with total photon flux (Figure S11b, Supporting Information). This indicates that glutamine competitively inhibits AA201 uptake, thereby reducing the bioluminescent signal.

Considering the structural homology between AA201 and glutamine, we hypothesized potential transport by alternative amino acid transporters. To test this, we employed BLGLN to systematically assess the effects of selective transporter inhibitors: JPH203 (LAT1 inhibitor), MeAIB (SNATs inhibitor), and α‐MT (ATB^0,+^ inhibitor).^[^ ^39^ ^]^ Bioluminescence intensity revealed exclusive inhibition by α‐MT (**F**igure S12, Supporting Information), suggesting ATB^0,+^ may recognize AA201 as a substrate. Compared to glutamine, competitive inhibition of AA201, suppression of transport capacity resulted in inhibiting both the growth rate of BL intensity and BL intensity during the plateau phase (Figure 6e). However, kinetic profiling showed that while α‐MT caused transient inhibition, both the growth rate of BL intensity and BL intensity during the plateau phase ultimately reached levels of the control group (Figure S12, Supporting Information). These data indicate that ATB^0,+^ plays only a minor role in AA201 transport, with ASCT2 serving as the primary transporter.

**Figure 7:**
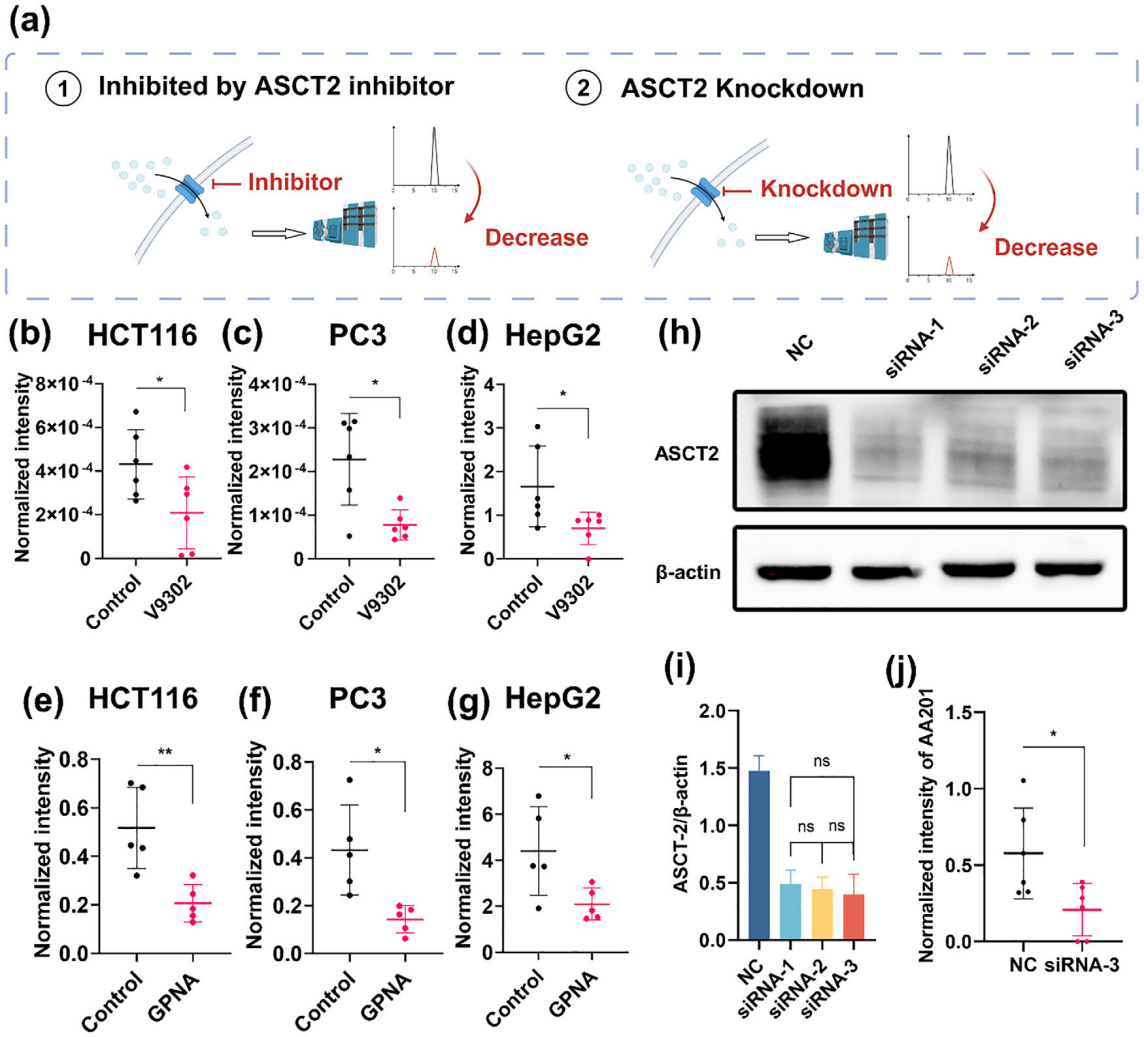
Validation of ASCT2‐mediated uptake of AA201 into tumor cells. a) Using ASCT2 inhibitors and ASCT2 knockdown to block the uptake of AA201. Determination of intracellular AA201 content in b) HCT116 c) PC3 d) HepG2 cells pretreated or non‐pretreated by V9302, *n *= 6. Determination of intracellular AA201 content in e) HCT116 f) PC3 g) HepG2 cells pretreated or non‐pretreated by GPNA, *n* = 5. h) Detection of ASCT2 expression levels by Western Blot. i) Grayscale value analysis of (h), *n *= 3. j) Determination of intracellular AA201 content in negative control HCT116 cells and ASCT2 knockdown HCT116 cells. ns *p* > 0.05, *p < 0.05, **p < 0.01.

To further corroborate ASCT2's role as the primary transporter mediating cellular internalization of AA201, we employed two complementary strategies—ASCT2 inhibitor treatment and expression knockdown—coupled with UPLC‐QQQ‐MS analysis for relative quantification of intracellular AA201 level in tumor cells (**Figure** **7**a). Initially, V9302 was used to assess the uptake mechanism in three tumor cell lines: HCT116, PC3 (prostate cancer), and HepG2 (liver cancer). Compared to untreated cells, V9302 treatment significantly reduced intracellular AA201 levels in all three cell lines (HCT116: Figure 7b; PC3: Figure 7c; HepG2: Figure 7d). To corroborate these findings, a parallel experiment was conducted using GPNA, another ASCT2 inhibitor. Consistent results demonstrated that GPNA treatment also significantly decreased AA201 uptake in all three tumor cell lines (HCT116: Figure 7e; PC3: Figure 7f; HepG2: Figure 7g). These consistent experimental observations indicate that ASCT2 inhibitors effectively suppress AA201 uptake by tumor cells.

Acknowledging the presence of multiple glutamine transporters in tumor cells and the limited specificity of current ASCT2 inhibitors, we employed an expression knockdown strategy to further validate ASCT2's role in AA201 uptake. Three distinct siRNA sequences were designed and transfected into HCT116 cells to suppress ASCT2 expression. Western blot analysis confirmed effective ASCT2 knockdown by all three siRNAs (Figure 7h,i), with no statistically significant differences. Consequently, all three siRNAs were equally effective in reducing ASCT2 expression, and one of the siRNAs (siRNA‐3) was selected for subsequent experiments. UPLC‐QQQ‐MS analysis revealed a significant reduction in intracellular AA201 content in the siRNA‐3‐transfected group compared to the negative control (Figure 7j). These results further confirm ASCT2 as the primary transporter responsible for AA201 internalization, indicating that AA201 serves as a reliable probe for evaluating ASCT2‐mediated glutamine uptake.

### 2.5 Investigating Glutamine Uptake Dynamics in Tumor Cells by BLGLN‐Based Real‐Time Imaging

**Figure 8:**
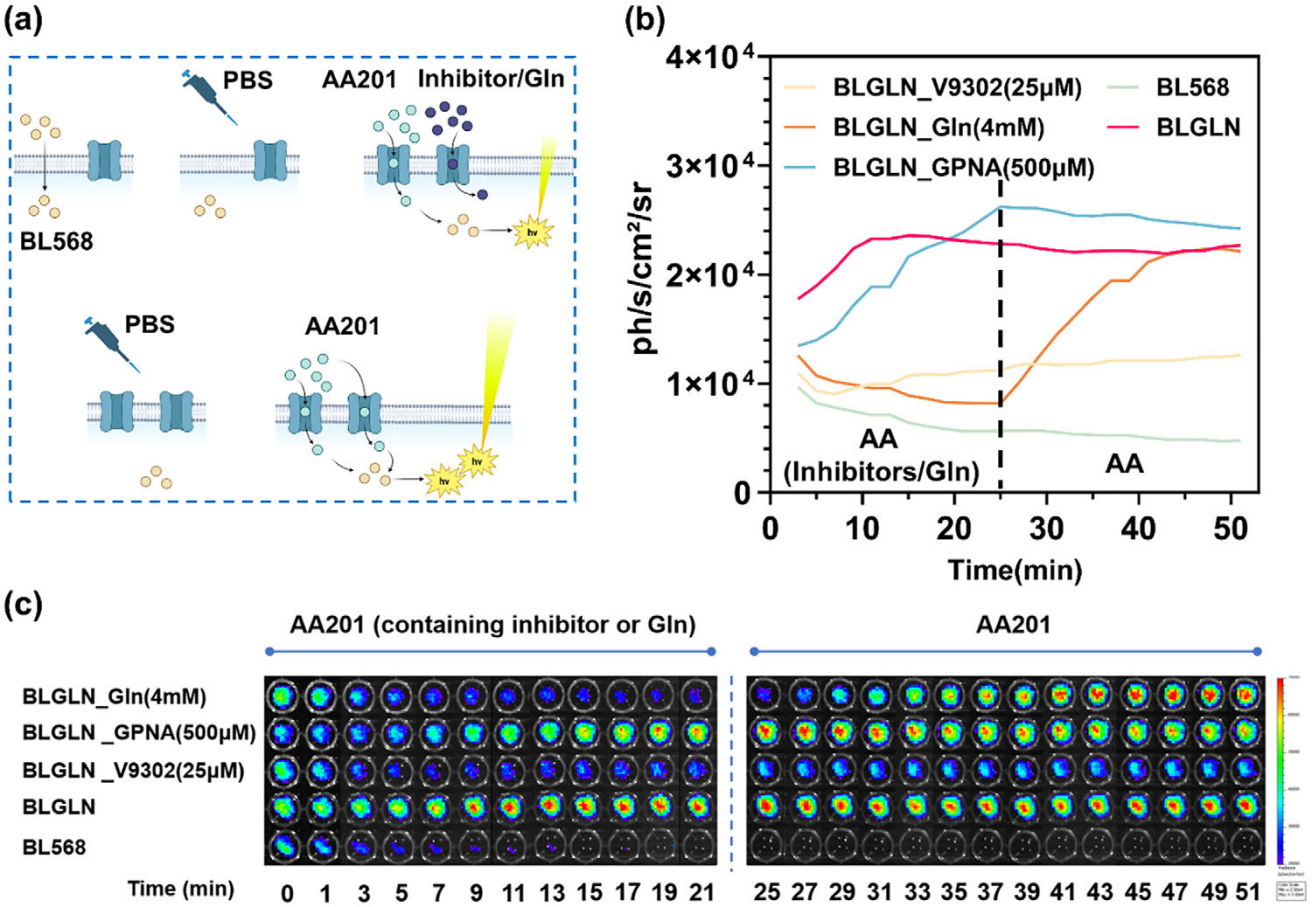
Investigating glutamine uptake dynamics in tumor cells by BLGLN‐based real‐time imaging. a) Scheme for validating the real‐time monitoring ability of the BLGLN. b) BL intensity time course plot. The part before the dashed line indicates the addition of AA201 solution containing different competitive inhibitors. The phase after the dashed line indicates that the solutions are replaced by the AA201 solution. *n* = 6. c) Visual monitoring images of (b).

To assess BLGLN's capacity to visually monitor dynamics in tumor cell glutamine uptake in vitro, a reverse inhibition experiment was designed (**Figure** **8**a). The experiment consisted of two phases: in the first phase, cells pre‐incubated with BL568 were treated with AA201 solutions containing ASCT2 inhibitors (V9302 or GPNA) or glutamine, and bioluminescent signals were monitored for 20 min. In the second phase, the solution was replaced with an inhibitor‐free AA201 solution, and bioluminescent signal changes were continuously monitored. Experimental results demonstrated that during the first phase, the BLGLN_V9302 (25 µM) and BLGLN_Gln (4 mM) groups exhibited low bioluminescent intensity, while the BLGLN_GPNA (500 µM) group showed slower signal growth, eventually reaching levels comparable to the BLGLN group. Upon solution replacement, the BLGLN_GPNA (500 µM) and BLGLN groups maintained high‐intensity signals, the BLGLN_V9302 (25 µM) group showed a gradual signal increase, and the BLGLN_Gln (4 mM) group rapidly recovered to the BLGLN group's intensity (Figure 8b). Visual representations directly illustrate the dynamics in tumor cell glutamine uptake (Figure 8c). Statistical analysis of total photon flux during the stable phase (41–51 min, Figure S13, Supporting Information) revealed a significant increase in total photon flux in the BLGLN_Gln (4 mM) group during the second phase compared to the first phase (11–21 min, Figure S13, Supporting Information), with no significant difference from the BLGLN group. This indicates that BLGLN can effectively monitor dynamics in tumor cell glutamine uptake, particularly the rapid recovery of uptake capacity following glutamine removal. These results comprehensively demonstrate the probe's ability to visually and in real‐time monitor dynamics in ASCT2‐mediated glutamine uptake in tumor cells.

### 2.6 In Vivo Imaging and Detection of Glutamine Uptake in Tumor by BLGLN

**Figure 9:**
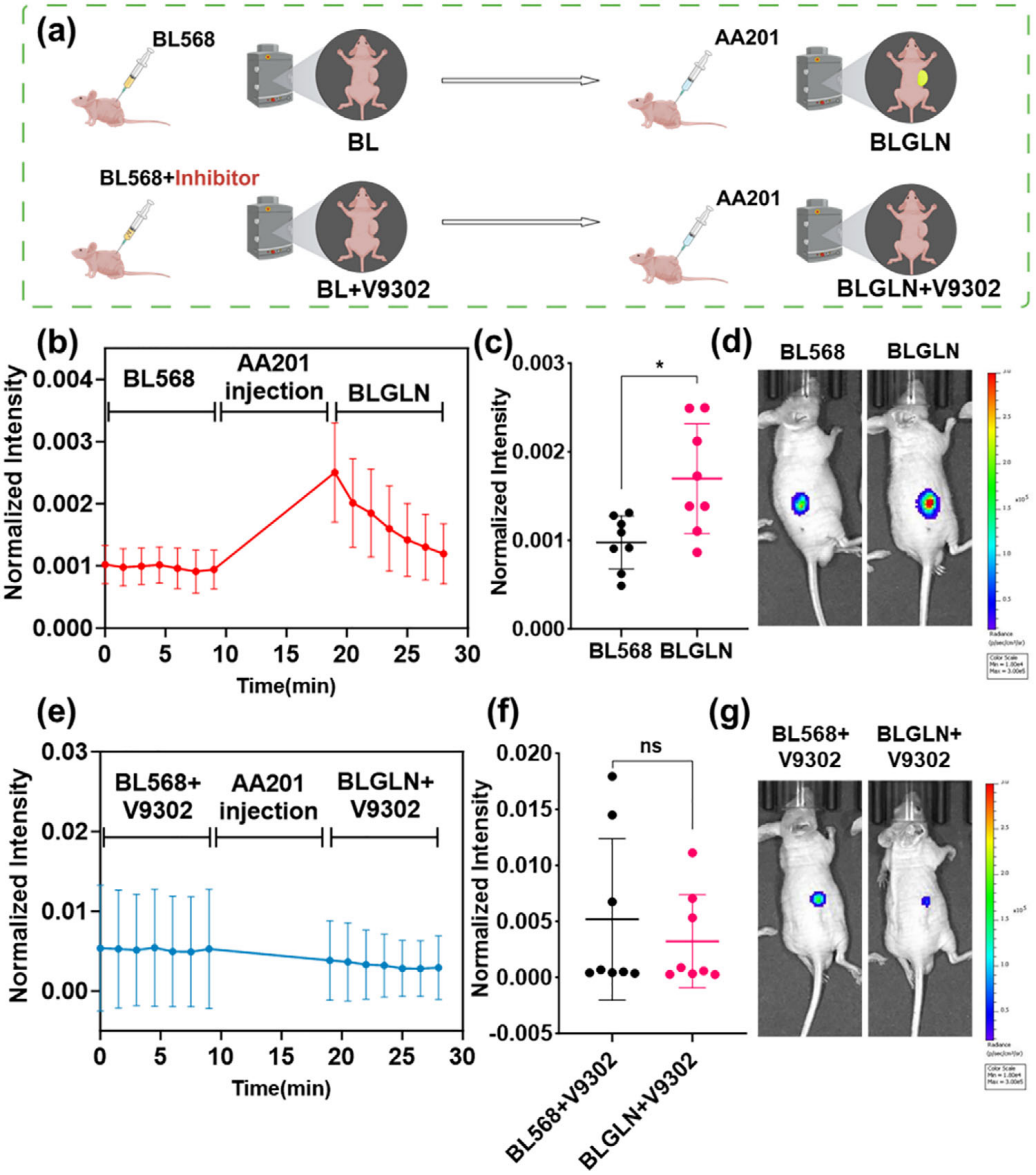
In vivo imaging and detection of glutamine uptake in tumors by BLGLN. a) In vivo bioluminescent imaging processes. Bioluminescent normalized intensity time course plot of b) BL568_BLGLN group and e) BL568+V9302_BLGLN+V9302 group. Bioluminescent normalized intensity average value of c) BL568_BLGLN group and f) BL568+V9302_BLGLN+V9302 group in the detecting period. Bioluminescent images of d) BL_BLGLN group and g) BL+V9302_BLGLN+V9302 group. *n *= 8. ns *p *> 0.05, *p < 0.05.

Following validation of the BLGLN's in vitro dynamic monitoring and glutamine uptake rate evaluating performance, its potential for in vivo applications was assessed. A subcutaneous tumor model was established in male BALB/c Nude mice using HCT116‐Luc cells (tumor volume ≈45 mm^3^). BL568 solution was intratumorally injected, and initial imaging was performed 3 h postinjection. Subsequently, the AA201 solution was injected, and real‐time imaging was immediately conducted (**Figure** **9**a). Results showed minimal signal detection during the BL568 injection phase, while a significant increase in bioluminescent signal at the tumor site was observed after AA201 injection, albeit with rapid signal decay (Figure 9b). Mean bioluminescent intensity analysis confirmed a significant increase in average intensity post‐AA201 injection (*p* < 0.05, Figure 9c). Representative imaging visually illustrated these signal changes (Figure 9d). To evaluate BLGLN's ability to detect in vivo glutamine uptake changes, the V9302 inhibitor was intratumorally injected 2 h post‐BL568 injection (Figure 9a), with subsequent imaging steps maintained. Imaging results showed no significant signal enhancement in the V9302‐treated group, either before or after AA201 injection (Figure 9e). Mean bioluminescent intensity analysis confirmed no significant difference between the BL568+V9302 and BLGLN+V9302 groups (*p* > 0.05, Figure 9f). Representative imaging visually demonstrated the absence of signal enhancement (Figure 9g). These in vivo experiments demonstrate that BLGLN effectively monitors and quantifies tumor cell glutamine uptake both in vitro and in vivo.

## 3 Conclusion

Metabolic reprogramming represents a hallmark of tumorigenesis, wherein “glutamine addiction” emerges as a critical metabolic dependency that sustains multiple malignant phenotypes. ASCT2 (SLC1A5), the principal transporter responsible for cellular glutamine uptake, is markedly upregulated across diverse malignancies to meet their increased glutamine demands. This upregulation critically supports tumor survival, progression, and therapeutic resistance by sustaining glutamine‐dependent metabolic pathways. Consequently, targeting ASCT2‐mediated glutamine uptake has emerged as a promising anti‐tumor strategy. Therefore, the ability to monitor ASCT2‐mediated glutamine transport is crucial for both investigating tumor metabolism and developing novel ASCT2‐targeted therapeutics. However, current methods for glutamine uptake detection are generally limited by complex sample preparation procedures and/or an inability to reliably quantify and real‐time monitor dynamics in glutamine uptake both in vitro and in vivo. In this work, we report BLGLN, an innovative bioluminescent probe system employing Staudinger ligation to enable real‐time monitoring of ASCT2‐mediated glutamine uptake dynamics in live tumors. BLGLN offers several key advantages: 1) simplified synthesis and preparation; 2) elimination of complex sample preparation procedures; and 3) real‐time monitoring and evaluation of glutamine uptake rate both in vitro and in vivo.

The successful development of BLGLN addresses the current technological deficiency in optical imaging methodologies for accurate tumor glutamine uptake assessment, presenting significant application potential: 1) providing a robust analytical tool for tumor glutamine metabolism research; 2) facilitating high‐throughput screening of ASCT2 inhibitors; and 3) serving as a foundational design for developing advanced optical probes with enhanced signal‐to‐noise ratios and tissue penetration for tumor glutamine uptake monitoring. This advancement not only contributes to the evolution of tumor metabolism research technologies but also offers a novel tool for real‐time monitoring and evaluation in the development of anti‐tumor strategies targeting glutamine uptake

## 4 Experimental Section

### Chemical and Materials

All commercially available reagents and solvents were used as received unless stated otherwise. Reaction progress was determined via TLC (Liangchen GuiYuan, HPTLC GF254) via UV visualisation (λ = 254 nm) and Phosphomolybdic acid staining. All column chromatography purifications were performed using analytic grade solvents (Sinopharm Chemical Reagent Co., Ltd.) and silica gel (Yantai Jiangyou Silica gel development Co., LTD, 200–300 mesh). Boc‐L‐2,4‐diaminobutyric acid, azidoacetic acid, and TSTU were purchased from Shanghai Haohong Biomedical Technology Co., Ltd. DMAP and DIPEA were purchased from Shanghai Aladdin Biochemical Technology Co., Ltd. V9302 and GPNA were purchased from MCE.

Acetonitrile (ACN, LC‐MS grade) and methanol (MeOH, LC‐MS grade) were supplied by Merck (Darmstadt, Germany). Ammonium formate was purchased from Sigma‐Aldrich (St. Louis, MO, USA). Fetal bovine serum (FBS) was purchased from BI (Biological Industries Israel Beit Haemek Ltd., Israel). Dulbecco's modified Eagle's medium (DMEM) was purchased from Guangzhou Yike Biotechnology Co., Ltd. (Guangzhou, China). Phosphate‐buffered saline (PBS) was obtained from Zhejiang Senrui Biotechnology Co., Ltd. (Hangzhou, China). siRNAs were purchased from Suzhou GenePharma Co., Ltd., the primary antibody was purchased from Proteintech Group (Chicago, USA), and the secondary antibodies were purchased from Abcam (Cambridge, UK).

### Instruments

The instruments used in this study including high‐resolution mass spectrometry (HR‐MS) with an UHPLC LTQ Orbitrap Elite mass spectrometer (ThermoScientific, Bremen, Germany), triple quadrupole mass spectrometry (QQQ‐MS) with an AB Sciex QTRAP 5500+ mass spectrometer (AB Sciex, USA), nuclear magnetic resonance spectroscopy (NMR) with an AVANCE III 500 MHz spectrometer (Bruker, Germany), multifunctional microplate reader with an INFINITE 200 (TECAN, Switzerland), small animal in vivo imaging system with an IVIS Spectrum (PerkinElemer, USA), and semi‐prep HPLC with a LC‐100 (Wufeng, China).

### Synthesis and Characterization—Compound 464

Weigh out 240 mg (1 equivalent, 0.78 mmol) of 2‐(diphenylphosphino)benzoic acid, 169.6 mg (1.1 equivalents, 0.82 mmol) of EDCI, and 4.7 mg (0.05 equivalents, 0.04 mmol) of DMAP, and dissolve them in 10 mL of DCM. Stir the mixture at room temperature for 30 min. Then, weigh out 144.83 mg (1.05 equivalents, 0.82 mmol) of 6‐hydroxybenzothiazole‐2‐carbonitrile and add it to the reaction mixture. Continue stirring at room temperature and monitor the reaction by TLC (DCM:MeOH = 20:1). Terminate the reaction when the 2‐(diphenylphosphino)benzoic acid in the reaction mixture is completely consumed. Remove the solvent by reduced pressure distillation and purify the product using silica gel column chromatography (PE:EA = 8:1) to obtain Compound 464 as a pale yellow oily liquid (242 mg, yield 67%). TLC analysis (DCM:MeOH = 20:1, Rf = 0.95) confirms the product. ^1^H‐NMR (500 MHz, CDCl_3_) δ 8.30–8.24 (m, 1H), 8.15 (d, J = 9.0 Hz, 1H), 7.55 (d, J = 2.2 Hz, 1H), 7.53–7.47 (m, 2H), 7.40–7.27 (m, 10H), 7.23 (dd, J = 9.0, 2.3 Hz, 1H), 7.03 (ddd, J = 7.2, 3.9, 2.0 Hz, 1H). ^13^C‐NMR(126 MHz, CDCl_3_) δ 165.01 (d, J = 2.5 Hz), 150.77 (s), 150.09 (s), 141.89 (s), 141.67 (s), 137.39 (d, J = 10.6 Hz), 136.74 (s), 136.11 (s), 134.72 (s), 134.27 (s), 134.10 (s), 133.14 (s), 132.80 (d, J = 18.3 Hz), 131.59 (d, J = 2.2 Hz), 129.10 (s), 128.81 (d, J = 7.4 Hz), 128.60 (s), 125.89 (s), 122.96 (s), 114.72 (s), 112.89 (s). MS (ESI+): m/z (C_27_H_17_N_2_O_2_PS): 465.0 [M+H]^+^.

### Synthesis and Characterization—BL568

Weigh out 242 mg (1 equivalent, 0.52 mmol) of the previous step product, Compound 464, and dissolve it in 6 mL of solvent (DCM:MeOH = 1:1). Weigh out 125.9 mg (2 equivalents, 1.04 mmol) of D‐cysteine and 143.7 mg (2 equivalents, 1.04 mmol) of potassium carbonate, and dissolve them in 6 mL of solvent (H2O:MeOH = 1:1). Subsequently, mix the two solutions and stir at room temperature for 10 min. Immediately remove the organic solvent from the reaction system by reduced‐pressure distillation. Adjust the pH of the reaction system to between 2 and 3 using 0.1 m HCl, and observe the formation of a precipitate. Filter to collect the precipitate and wash it with an appropriate amount of ice‐cold water. Dissolve the obtained precipitate in a suitable amount of methanol, and then perform separation and purification using RP‐HPLC. The RP‐HPLC purification conditions were as follows: flow rate of 2 mL min^−1^, column temperature of 25 °C, detection wavelength of 254 nm, and a chromatographic column (ACCHROM, C18 10 µm 10 × 250 mm). The mobile phase consisted of (A) water and (B) methanol. Remove the solvent by reduced pressure distillation to obtain the bioluminescent structure BL568 as a pale yellow powder (59 mg, yield 20%). ^1^H‐NMR(500 MHz, DMSO‐d_6_) δ 8.30–8.25 (m, 1H), 8.18 (d, J = 8.9 Hz, 1H), 7.90 (d, J = 2.2 Hz, 1H), 7.66–7.62 (m, 2H), 7.40 (d, J = 3.8 Hz, 6H), 7.27 (dd, J = 8.9, 2.3 Hz, 1H), 7.25–7.19 (m, 4H), 6.94 (dt, J = 5.2, 3.5 Hz, 1H), 5.45 (dd, J = 9.7, 8.5 Hz, 1H), 3.84–3.77 (m, 1H), 3.72 (dd, J = 11.2, 8.4 Hz, 1H). ^13^C‐NMR(126 MHz, DMSO‐d_6_) δ 171.03 (s), 164.73 (d, J = 1.9 Hz), 164.30 (s), 161.26 (s), 150.56 (s), 149.07 (s), 140.47 (s), 140.24 (s), 137.09 (d, J = 11.4 Hz), 135.98 (s), 133.94 (s), 133.64 (s), 133.47 (s), 133.15 (s), 132.85 (s), 131.23 (s), 129.04 (s), 128.81 (d, J = 7.2 Hz), 124.72 (s), 121.84 (s), 115.78 (s), 78.27 (s), 34.91 (s). ^31^P NMR (202 MHz, DMSO‐d_6_) δ ‐5.74 (s). HRMS (ESI+): m/z (C_30_H_21_N_2_O_4_PS_2_): 569.08337 [M+H]^+^ (Table 1).

**Table 1:** BL568 separation and purification mobile phase gradient table.

Time	Concentration of B Phase
0 min	40%
40 min	100%
55 min	100%
58 min	40%
68 min	40%

**Table 2:** UPLC‐QQQ‐MS Mobile Phase Gradient for AA201 Detection.

Time [min]	% Mobile Phase B
0	90
15	50
20	50
25	90
40	90

**Table 3:** Ion Source Parameters.

Parameter	Value
Curtain Gas(CUR)	35.0
Collision Gas(CAD)	9
IonSpray Voltage(IS)	−4500.0
Temperature(TEM)	350
Ion Source Gas 1(GS1)	45
Ion Source Gas 2(GS2)	50

**Table 4:** MRM Detection Parameters for AA201 and Internal Standard.

Compound	Q1 Mass[Da]	Q3 Mass[Da]	Dwell Time[msec]	DP[volts]	CE[volts]
AA201	200.0	172.0	100	−76.9	−11.96
	200.0	154.0	100	−76.9	−12.28
	200.0	117.0	100	−76.9	−18.01
Boc‐L‐2,4‐diaminobutyric acid	217.1	217.1	100	−6.92	−6.7
	217.1	143.0	100	−6.92	−11.12

**Table 5:** MRM Detection Parameters for [^13^C_5_, ^15^N_2_]‐L‐Glutamine and Internal Standard.

Compound	Q1 Mass[Da]	Q3 Mass[Da]	Dwell Time[msec]	DP[volts]	CE[volts]
[^13^C_5_, ^15^N_2_]‐L‐Glutamine	152.0	134.0	100	−56.0	−15.96
	152.0	116.0	100	−56.0	−18.18
	152.0	89.0	100	−56.0	−20.06
Boc‐L‐2,4‐diaminobutyric acid	217.1	217.1	100	−6.92	−6.7
	217.1	143.0	100	−6.92	−11.12

### Synthesis and Characterization—AA201

The synthesis of AA201 began by dissolving azidoacetic acid (0.8 mmol, 80.84 mg), TSTU (0.88 mmol, 264.92 mg), and DIPEA (1.04 mmol, 134.4 mg) in 10 mL of acetonitrile (ACN), followed by stirring at room temperature for 15 min. Boc‐L‐2,4‐diaminobutyric acid (0.88 mmol, 192.06 mg) was then dissolved in 10 mL of water and added to the reaction mixture, which was stirred at room temperature. The reaction progress was monitored by TLC (DCM:MeOH = 10:1) until the azidoacetic acid was fully consumed. After removing ACN under reduced pressure, the pH of the aqueous phase was adjusted to 10–11 using dilute NaOH solution, and the aqueous phase was washed with DCM (15 mL × 3). The pH of the aqueous phase was then adjusted to 1 using dilute HCl, and the mixture was extracted with ethyl acetate (EA, 15 mL × 3). The organic phases were combined, and the solvent was removed under reduced pressure to obtain compound 301. No further purification is required. The obtained crude product of compound 301 was dissolved in 10 mL of DCM, followed by the addition of 10 mL of a 4m HCl solution in 1,4‐dioxane. The mixture was stirred at room temperature for 2 h, after which the solvent was removed under reduced pressure to yield the crude product of AA201. The crude AA201 was dissolved in water and purified using RP‐HPLC. The RP‐HPLC purification conditions were as follows: flow rate of 1 mL min^−1^, column temperature of 25 °C, detection wavelength of 210 nm, and a chromatographic column (ACCHROM, C18 10 µm 10 × 250 mm). The mobile phase consisted of 95% water (A phase) and 5% ACN (B phase). The final obtained AA201 is a white solid (120 mg, yield 75%). ^1^H‐NMR (500 MHz, D_2_O) δ 4.06 (s, 2H), 3.74 (t, J = 6.5 Hz, 1H), 3.54–3.32 (m, 2H), 2.11 (dtt, J = 28.5, 14.2, 7.0 Hz, 2H). ^13^C‐NMR (126 MHz, D_2_O) δ 174.03 (s), 170.88 (s), 52.43 (s), 51.83 (s), 35.50 (s), 30.05 (s). HRMS (ESI+): m/z (C_6_H_11_N_5_O_3_): 200.07741 [M‐H]^−^.

### Synthesis and Characterization—Compound 234

A solution of N‐BOC‐1,4‐phenylenediamine (208.8 mg, 1.0 mmol, 1.0 eq) and DMAP (146.7 mg, 1.2 mmol, 1.2 eq) in acetonitrile (4 mL) was combined with a separately prepared solution of 2‐azido‐1,3‐dimethylimidazolinium hexafluorophosphate (344.6 mg, 1.2 mmol, 1.2 eq) in acetonitrile (2 mL). The resulting mixture was heated to 30 °C with stirring, and the reaction progress was monitored by TLC. Upon complete consumption of N‐BOC‐1,4‐phenylenediamine, the reaction was quenched with saturated aqueous NaHCO_3_ solution (20 mL) and extracted with dichloromethane (DCM) (3 × 15 mL). The combined organic layers were washed with saturated NaCl solution, dried over anhydrous Na_2_SO_4_, and concentrated under reduced pressure. The crude product was purified by flash column chromatography (DCM/MeOH = 100:1) to afford Compound 234 as a light yellow powder (136 mg, 58% yield). ^1^H NMR (500 MHz, CDCl_3_) δ 7.35 (d, J = 8.5 Hz,2H), 6.98 – 6.91 (m, 2H), 6.46 (s, 1H), 1.51 (s, 9H).

### Synthesis and Characterization—Compound 134

Compound 234 (21.2 mg, 0.09 mmol) was dissolved in methanol (1 mL), followed by the addition of 4 m HCl in 1,4‐dioxane (1 mL). The reaction mixture was stirred at room temperature for 60 min, with the reaction progress monitored by TLC. After complete consumption of the starting material, the solvent was removed under reduced pressure to afford the hydrochloride salt of Compound 134 as a white solid (15.1 mg, 98% yield). The product was used directly in the next step without further purification.

### Synthesis and Characterization—Compound 419

Boc‐L‐glutamic acid 1‐tert‐butyl ester (21.8 mg, 0.072 mmol, 1.2 eq), DMAP (36.6 mg, 0.3 mmol, 5 eq), and EDCI (23 mg, 0.12 mmol, 2 eq) were dissolved in DMSO (3 mL) and stirred at room temperature for 30 min. Subsequently, the hydrochloride salt of Compound 134 (11 mg, 0.065 mmol, 1 eq) was directly added to the reaction mixture, and stirring was continued at room temperature. The reaction progress was monitored by TLC. Upon complete consumption of Compound 134, the reaction was quenched by dilution with ethyl acetate, followed by extensive washing with water to remove residual DMSO. The organic phase was further washed with saturated NaCl solution, dried over anhydrous Na_2_SO_4_, and concentrated under reduced pressure. The crude product was purified by flash column chromatography (DCM/MeOH = 100:1) to afford Compound 419 as a pale yellow oil (16 mg, 58% yield). ^1^H NMR (500 MHz, CDCl_3_) δ 9.00 (s, 1H), 7.63 (d, J = 8.3 Hz, 2H), 6.98 (d, J = 8.8 Hz, 2H), 5.38 (d, J = 7.5 Hz, 1H), 4.21 (s, 1H), 2.46–2.39 (m, 2H), 2.31–2.21 (m, 1H), 1.90–1.79 (m, 1H), 1.46 (d, J = 6.8 Hz, 18H).

### Synthesis and Characterization—AA263

Compound 419 (16 mg, 0.038 mmol) was dissolved in dichloromethane (DCM) (1 mL), followed by the addition of 4 m HCl in 1,4‐dioxane (1 mL). The reaction mixture was stirred at room temperature while being monitored by LC‐MS (negative ion mode). The reaction was terminated when the characteristic signal at m/z = 319.2 disappeared completely. The solvent was then removed under reduced pressure to afford the hydrochloride salt of AA263 as a white solid (11 mg, 95% yield). The product was used directly in subsequent steps without further purification. ^1^H NMR (500 MHz, DMSO‐d_6_) δ 10.37 (s, 1H), 8.51 (d, J = 3.6 Hz, 4H), 7.81 (d, J = 2.0 Hz, 1H), 7.38 (dd, J = 8.7, 2.0 Hz, 1H), 7.19 (d, J = 8.6 Hz, 1H), 3.92 (dd, J = 11.1, 5.5 Hz, 1H), 2.70–2.56 (m, 2H), 2.10 (dt, J = 15.1, 7.0 Hz, 2H).

### Cells and Cell Culture

HCT116, PC3, HepG2 cell lines were purchased from the Cell Bank of the Chinese Academy of Sciences, Shanghai. PC3 cells and HCT116 cells were cultured in RPMI 1640 complete medium supplemented with 10% FBS. HepG2 cells were cultured in DMEM complete medium supplemented with 15% fetal bovine serum. The cells were incubated in a cell culture incubator under conditions of 5% CO_2_ at 37 °C.

### Detection of Intracellular AA201 Content by LC‐MS after Incubation with Inhibitors

The intracellular content of AA201 was detected by first harvesting cells from 10 cm culture dishes, centrifuging them at 800 rpm for 5 min, and resuspending the pellet in 2 mL of fresh complete medium. After counting the cells using a hemocytometer, they were seeded into a 6‐well plate at a density of 875000 cells per well and incubated for 24 h. The old medium was then replaced with glutamine‐free RPMI 1640 or DMEM complete medium (with or without V9302 (25 µM) or GPNA (250 µM)) for 1 h, followed by treatment with AA201 in glutamine‐free complete medium for 10 min. After discarding the medium, the cells were washed with PBS on ice, scraped in 500 µL of pre‐chilled 50% methanol, and collected into 1.5 mL centrifuge tubes. An additional 300 µL of 50% methanol was used for washing, and the combined solution was mixed with 360 µL of pre‐chilled chloroform, vortexed for 5 min, and then treated with 120 µL of pre‐chilled water, followed by vortexing for 2 min. The mixture was centrifuged at 12000 rpm for 10 min at 4 °C, and the supernatant was collected, dried using a SpeedVac, and stored at −80 °C. The remaining protein layer was air‐dried, lysed with 70 µL of RIPA buffer on ice for 20 min, and centrifuged at 12000 rpm for 10 min at 4 °C, with the supernatant used for protein concentration measurement via a BCA assay kit to normalize AA201 content. For UPLC‐QQQ‐MS analysis, samples were dissolved in 50% acetonitrile containing the internal standard Boc‐L‐2,4‐diaminobutyric acid, centrifuged at 12000 rpm for 10 min at 4 °C, and the supernatant was transferred to sample vials. The UPLC‐QQQ‐MS conditions included an Acclaim Trinity P2 HPLC column (ThermoScientific, 3 µm, 2.1×100 mm), a mobile phase gradient (phase A: 20 mM ammonium formate in water; phase B: acetonitrile), a flow rate of 0.2 mL min^−1^, a column temperature of 37 °C, and specific ion source and MRM parameters (Tables 2, 3, 4). Data were processed using SCIEX OS software to integrate peak areas for accurate quantification of AA201.

### Detection of Intracellular AA201 Content after Knocking down Expression of ASCT2

siRNA and negative control (NC) were dissolved in DEPC water to a concentration of 20 µM, aliquoted, and stored at −80 °C. For transfection reagent preparation, Solution A was prepared by mixing 150 µL Opti‐MEM with 4 µL RNAmax, while Solution B was prepared by mixing 150 µL Opti‐MEM with 4 µL siRNA or NC. Solution B was then added to Solution A, mixed gently, and incubated for 10 min at room temperature. HCT116 cells were seeded into 6‐well plates at a density of 150000 cells per well and incubated until reaching 30%‐50% confluency. The culture medium was aspirated and replaced with 2 mL of fresh medium, followed by the addition of 308 µL of the prepared transfection reagent mixture to each well. After 48 h of incubation in a cell culture incubator, the expression levels of ASCT2 were analyzed to evaluate the knockdown efficiency. The siRNA sequences used for ASCT2 knockdown were as follows: ASCT2‐1 (sense: GCCUUGGCAAGUACAUUCUdTdT, antisense: AGAAUGUACUUGCCAAGGCdTdT), ASCT2‐2 (sense: CCGGUCCUGUACCGUCCUCAA, antisense: UUGAGGACGGUACAGGACCGG), and ASCT2‐3 (sense: CCUGGGCUUGGUAGUGUUUtt, antisense: AAACACUACCAAGCCCAGGtt). The method for subsequent detection of intracellular AA201 was the same as described 4.5.

### Western Blot

After 48 h of transfection, cells were scraped off, collected, and centrifuged at 800 rpm for 3 min at 25 °C. The supernatant was discarded, and the cell pellet was resuspended in PBS, centrifuged again, and lysed with 70 µL of RIPA lysis buffer on ice for 20 min. The lysate was centrifuged at 12000 rpm for 10 min at 4 °C, and the supernatant was used for protein concentration measurement via the BCA assay. A 40 µL aliquot of the supernatant was mixed with 10 µL of loading buffer, boiled at 95 °C for 5 min, and stored at −20 °C. For electrophoresis, 5 µL of protein marker and 20 µg of protein samples were loaded onto the gel, and electrophoresis was performed at 70 V for 30 min followed by 110 V for 70 min. The gel was then soaked in transfer buffer, and proteins were transferred to a methanol‐activated PVDF membrane at 250 mA for 70 min. After transfer, the membrane was washed three times with TPBS and blocked with 5% skim milk for 2 h. The membrane was cut according to the molecular weights of ASCT2 and β‐actin, incubated overnight at 4 °C with primary antibodies (diluted by antibody diluent, ASCT2 (1:8000), β‐actin (1:5000)), washed, and then incubated with secondary antibodies (diluted by TPBS, 1:5000) at room temperature for 2 h. Finally, the ASCT2 band was incubated with ECL detection reagent for 25 s, and the β‐actin band was incubated for 5 s, both imaged using a Bio‐Rad gel imaging system, and analyzed using ImageJ software.

### Molecular Docking

Molecular docking simulations were performed using the software Maestro 11.9. The protein data of ASCT2 (PDB ID: 6GCT, ligand: glutamine) were downloaded from the PDB database, and the receptor protein was processed by removing water molecules and ligands, adding hydrogen atoms, balancing charges, and minimizing free energy. The ligand structure of AA201 and AA263 was imported and prepared for docking. A docking box was generated for the receptor protein, and molecular docking was performed using the XP algorithm. After docking, the Docking Score and MM‐GBSA values were calculated. Visualization and rendering of the results were carried out using PyMOL software.

### Cell Viability Assay

For the cell viability assay, HCT116 cells were seeded into 96‐well plates (Corning, USA) at a density of 5×103 cells per well. Following an overnight incubation period, the cells were exposed to various concentrations of BL568 or AA201 for 24 h. BL568 was prepared as a 10 mM stock solution using DMSO, and then diluted to the desired concentrations with complete culture medium, ensuring that the concentration of DMSO was equal across all dilutions. AA201 was prepared to the desired concentrations using the complete culture medium. After the 24‐h treatment, cell viability was assessed using a CCK‐8 solution (Meilunbio, Dalian, China). The absorbance was measured at 450 nm using a Multiskan FC multimode microplate reader (ThermoFisher Scientific, USA). Background absorbance from wells containing only culture media was subtracted, and the absorbance readings were normalized relative to untreated cells.

### Construction of the HCT116‐Luc Cell Line

One day before infection, HCT116 cells were seeded into a 6‐well plate at a density of 150 000 cells per well and cultured for 24 h. After 24 h, 95 µL of RPMI 1640 medium was transferred to a 1.5 mL centrifuge tube, and 2.5 µL of the thawed pCDH‐MSCV‐MCS‐EF1‐copLuc‐T2A‐Puro virus solution, along with 2.5 µL of ADV‐HR (an infection‐enhancing reagent), were added to prepare the virus‐containing infection medium. HCT116 cells were trypsinized, centrifuged, and resuspended in the virus‐containing medium, followed by incubation at 37 °C for 30 min. The cells were then resuspended in 2 mL of complete medium and returned to the incubator. Fluorescence was monitored under an inverted microscope within 24‐h postinfection to assess infection efficiency. Once cell growth stabilized, puromycin was added at a concentration of 0.1 µg mL^−1^, and the concentration was gradually increased for selection until the transfection efficiency exceeded 80%, resulting in the successful generation of HCT116 cells stably expressing luciferase (HCT116‐Luc). For validation, HCT116‐Luc cells were seeded into black, clear‐bottom 96‐well plates at 40000 cells per well and cultured for 24 h. The medium was then discarded, and the cells were washed twice with PBS. KRH buffer or KRH buffer containing 25 µM D‐luciferin sodium was added, and the cells were incubated for 10 min before measuring the bioluminescence signal intensity using a multifunctional microplate reader. This protocol ensures the construction and validation of the HCT116‐Luc cell line for bioluminescence‐based assays.

### In Vitro Tumor Cells Glutamine Uptake Monitoring

BL568 was prepared as a 10 mM stock solution using DMSO and diluted to the desired concentration with complete medium. AA201 was diluted to the required concentration using KRH buffer. HCT116‐luc cells were seeded into a transparent‐bottom black 96‐well plate (Beyotime, China) at 40 000 cells per well and cultured in an incubator for 24 h. After 24 h, the old medium in the wells was aspirated, and 100 µL of BL568 solution was added to each well and incubated with the cells for 60 min. The complete medium containing BL568 was then removed, and the cells were washed twice with 200 µL of PBS. Subsequently, 100 µL of AA201 solution was added to each well, and the plate was immediately placed into a small animal in vivo imaging system or a multifunctional microplate reader to monitor the bioluminescence signal of each well. Use IVIS Spectrum software to analyze the bioluminescence signal intensity of each well. The cellular uptake rate was determined using the following normalized metric: uptake rate = (total photon flux _(experiment)_ – Total photon flux _(BL568)_)/(Total photon flux _(control)_ – Total photon flux _(BL568)_).

### The Selectivity of BL568 Toward Biorelevant Interfering Analytes

BL568 was prepared as a 10 mM stock solution using DMSO and diluted to 100 µM with DMSO. Biorelevant interfering analytes were prepared at double concentration in PBS, placing the prepared solution on ice. Mix BL568 solution and the Biorelevant interfering analytes solution in equal volumes and incubate at room temperature for 30 min. After incubation, the fluorescence intensity at 530 nm was measured, excited by 310 nm. Normalization intensity calculation formula: Normalized intensity = (F_Analytes_‐F_H2O_)/(F_AA201_‐F_H2O_)

### Detection of Intracellular [^13^C_5_, ^15^N_2_]‐L‐Glutamine Level by LC‐MS

The detection method is the same as 4.5. MRM parameters were adjusted (Table 5).

### Mice

Male BALB/c Nude mice, aged 3–5 weeks (SPF) were purchased from Jiangsu GemPharmatech Co., Ltd.

### Xenograft Tumor

BALB/c Nude male mice aged 3–5 weeks were used for the study. HCT116‐luc cells were cultured, harvested by trypsinization, and centrifuged to remove the supernatant. The cell pellet was resuspended in PBS to prepare a cell suspension at a concentration of 1×10^8^ cells mL^−1^. Two to three days after the mice were housed in the animal experiments center, 50 µL of the cell suspension was injected subcutaneously into each mouse using a 1 mL sterile syringe. Tumor growth was monitored daily, and when the tumor volume reached 45 mm^3^, the mice were ready for use. The mice that completed the modeling were then grouped randomly.

### In Vivo Tumor Glutamine Uptake Imaging and Detection

The 1.5 mM BL568 suspension was prepared by dissolving BL568 in PBS containing 0.1% BSA to achieve the desired concentration. The AA201 solution was prepared by dissolving AA201 in PBS to reach a concentration of ≈4.14 mg mL^−1^. The V‐9302 suspension was prepared at a concentration of 25 mg mL^−1^. For the preparation of BL568 and V‐9302, ultrasound was used to assist in the dissolution process.

The BL568 suspension was intratumorally injected, and 3 h later, the mice were anesthetized and placed into the small animal in vivo imaging system to monitor the bioluminescence signal for 10 min. Subsequently, the mice were removed from the imaging system, and the AA201 solution was intratumorally injected. Immediately after the injection, the mice were anesthetized again and placed back into the small animal in vivo imaging system to monitor the bioluminescence signal for another 10 min.

For the inhibitor group, the BL568 suspension was first intratumorally injected. Two hours later, the V9302 suspension was intratumorally injected. Three hours after the BL568 suspension injection, the mice were anesthetized and placed into the small animal in vivo imaging system to monitor the bioluminescence signal for 10 min. Subsequently, the mice were removed from the imaging system, and the AA201 solution was intratumorally injected. Immediately after the injection, the mice were anesthetized again and placed back into the small animal in vivo imaging system to monitor the bioluminescence signal for another 10 min.

Twenty‐four hours after completing the imaging, D‐luciferin (15 mg mL^−1^) was intraperitoneally injected at a volume of 100 µL per mouse. The data obtained from this procedure were used to normalize the bioluminescence signal intensity of the probe.

Use IVIS Spectrum software to analyze the bioluminescence signal intensity at the tumor site.

### Data Statistics and Analysis

The numerical values of the experimental results are expressed as mean ± standard deviation. Use SPSS to test the normality and homogeneity of variance of the data in each group. Employ the Bonferroni test of one‐way ANOVA to calculate the *p*‐value. For data that meet the normality but do not satisfy the homogeneity of variance, use the Tamhane test of one‐way ANOVA. For data that meet the normality, a *p*‐value less than 0.05 indicates a statistically significant difference, which is represented by “*”; a *p*‐value less than 0.01 indicates a significant statistical difference, represented by “**”; and a *p*‐value less than 0.001 indicates an extremely significant statistical difference, represented by “***”. The statistical graphs are completed using GraphPad Prism 10.1.2 software.

### Animal Ethics and Welfare

The animal ethical and welfare is approved by the Animal Ethical and Welfare Committee of HZNU (APPROVAL No. HSD‐20240423‐01).

## Conflict of Interest

The authors have no conflict of interest to declare.

## Supporting information



Supporting Information

## Data Availability

Research data are not shared.
