# Supplementary material for: Real‐Time Tracking of ASCT2‐Mediated Glutamine Uptake in Living Tumors With a Bioorthogonal Bioluminescent Probe
Source: Adv Sci (Weinh). 2025 Aug 10;12(40):e07057. doi: 10.1002/advs.202507057 (PMC12561342; doi:10.1002/advs.202507057)
Supplement: Supplementary file 1 — Supporting Information [file ADVS-12-e07057-s001.pdf]

Supporting Information

Real-Time Tracking of ASCT2-Mediated Glutamine Uptake in Living Tumors with a Bioorthogonal Bioluminescent Probe

Bing-Jun Zhou[a, b, c]*, Ji-Lei Zhao*[a, b]*, Fan Yin*[a, b]*, Wen-Da Chen*[a, b]*, Yi Sun*[a, b]*, Ying-Ying Ren*[a, b]*, Yi-Min Chen*[a, b]*, Tian Xie*[a, b]**, Chong Duan*[a, b]**, Jian-Liang Zhou*[a, b]***

[a] School of Pharmacy, Hangzhou Normal University, Hangzhou 311121, China
[b] Zhejiang Provincial Key Laboratory of Anti-Cancer Chinese Medicines and Natural Medicines, Hangzhou Normal University, Hangzhou, China
[c] Innovation Research Institute of Traditional Chinese Medicine, Shanghai University of Traditional Chinese Medicine, Shanghai 201203, China

***Corresponding Authors:**
Tian Xie,
Tel.: +86-571-28868269;
E-mail: tianxie@hznu.edu.cn

Chong Duan,
Tel.: +86-571-28860237;
E-mail: duanchong@hznu.edu.cn

Jian-Liang Zhou,
Tel.: +86-571-28860237;
E-mail: cpuzhou@hznu.edu.cn, cpuzhou@163.com

Content

Figure S1. 4

Figure S2. 6

Figure S3. 7

Figure S4. 8

Figure S5. 8

Figure S6. 9

Figure S7. 9

Figure S8. 10

Figure S9. 10

Figure S10. 11

Figure S11. 11

Figure S12. 12

Figure S13. 12

(a)

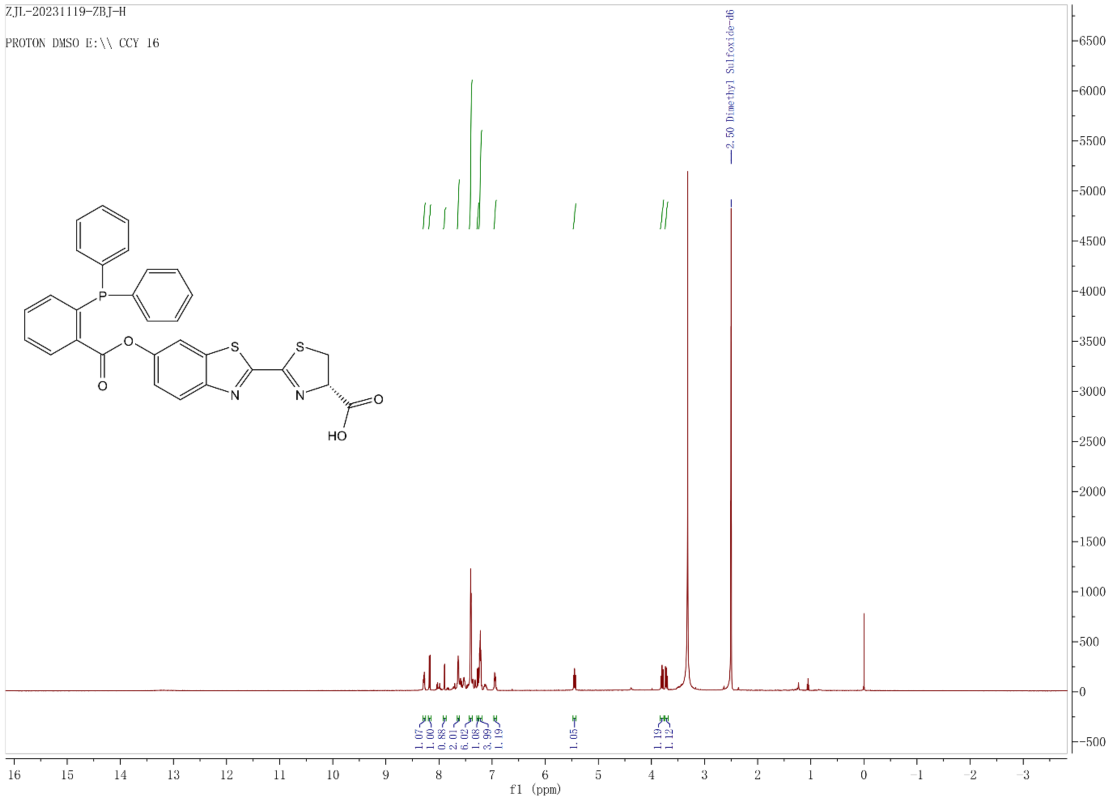
ZJL-20231119-ZBJ-H
PROTON DMSO E:\\ CCY 16
2.50 Dimethyl Sulfoxide-d6
f1 (ppm)

(b)

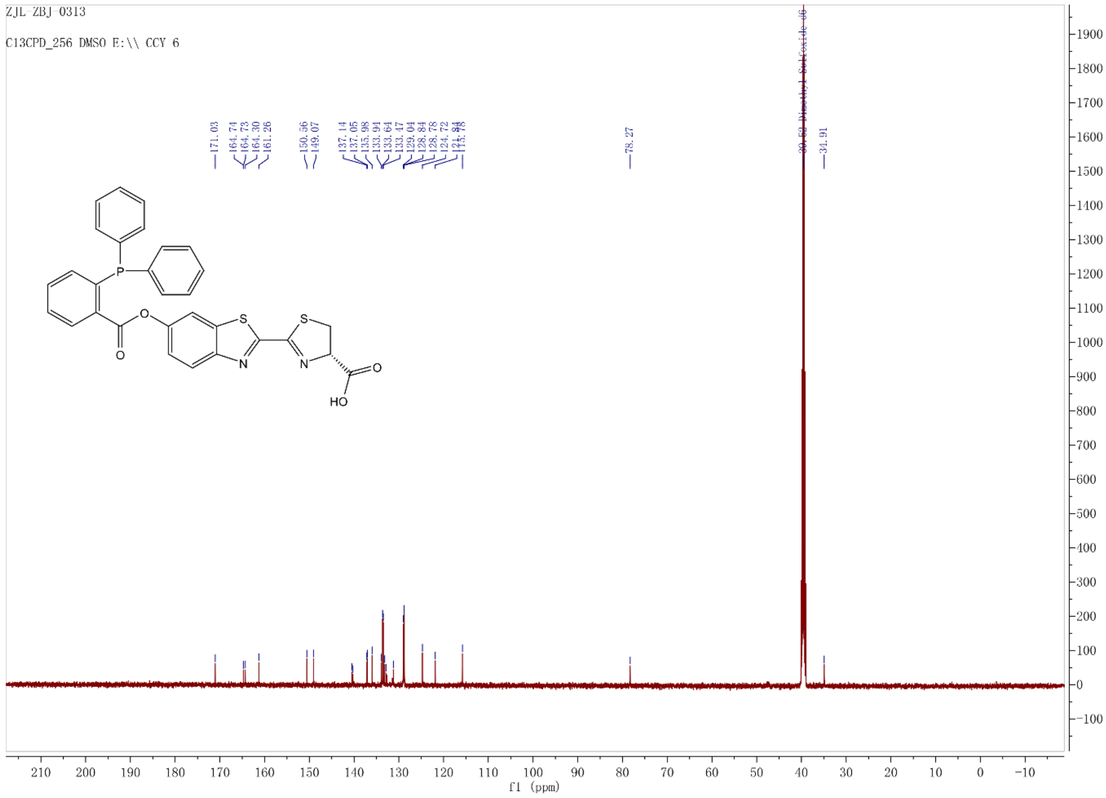
ZJL ZBJ 0313
C13CPD_256 DMSO E:\\ CCY 6
171.03
78.27
34.91
f1 (ppm)

(c)

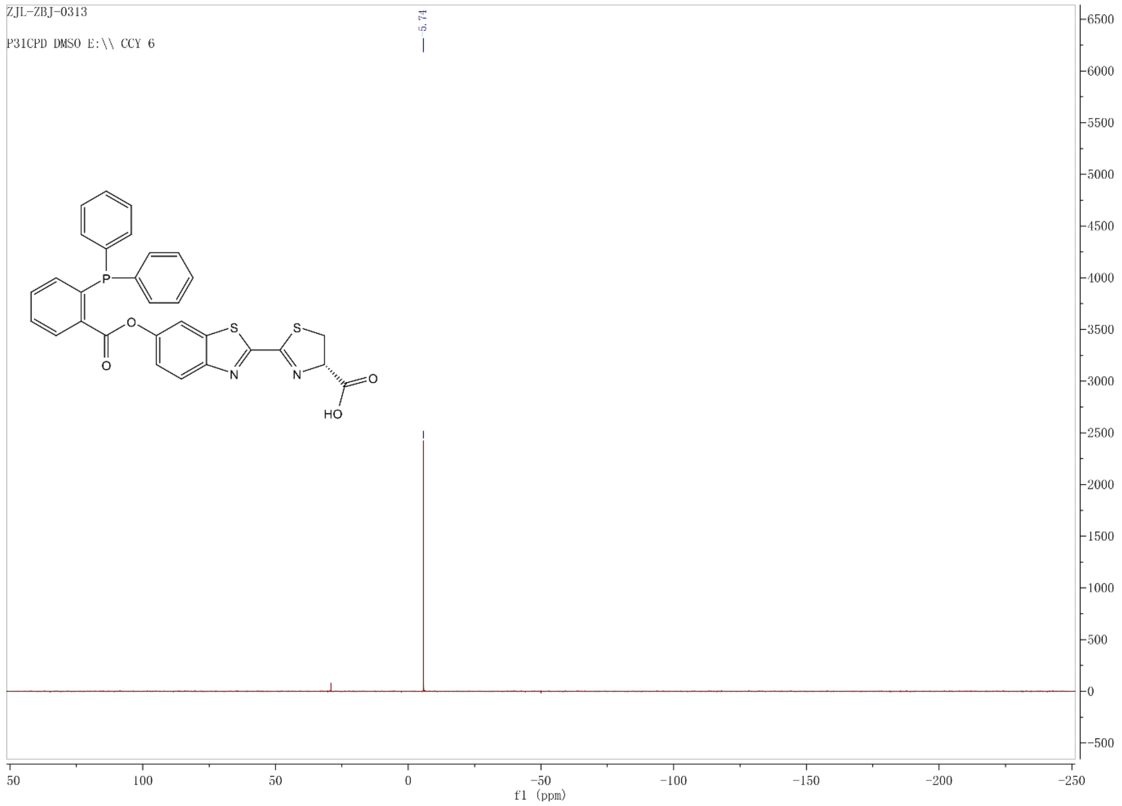

(d)

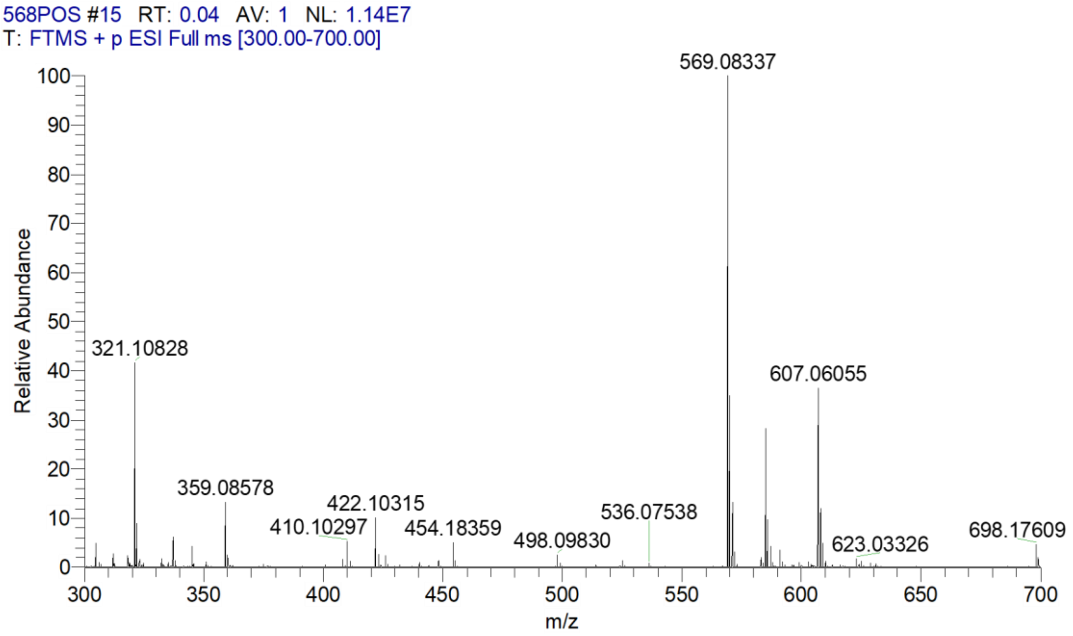

Figure S1. Structural characterization of BL568. (a) ^{1}H-NMR of BL568. (b) ^{13}C-NMR of BL568. (c) ^{31}P-NMR of BL568. (d) High-resolution mass spectrum of BL568.

(a)

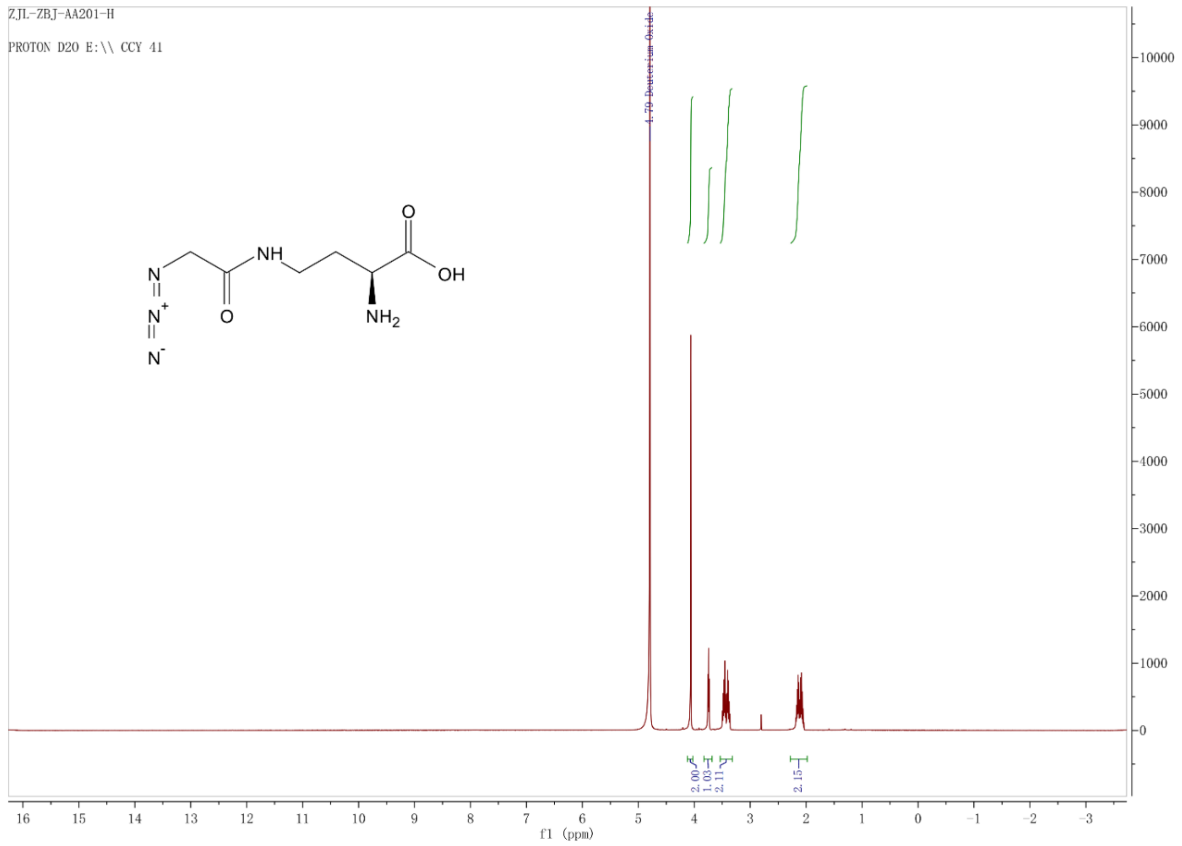

ZJL-ZBJ-AA201-H
PROTON D2O E:\\ CCY 41
4.79 Deuterium Oxide
O
NH
OH
N
N+
N-
O
NH2
2.00
1.03
2.11
2.15
f1 (ppm)

(b)

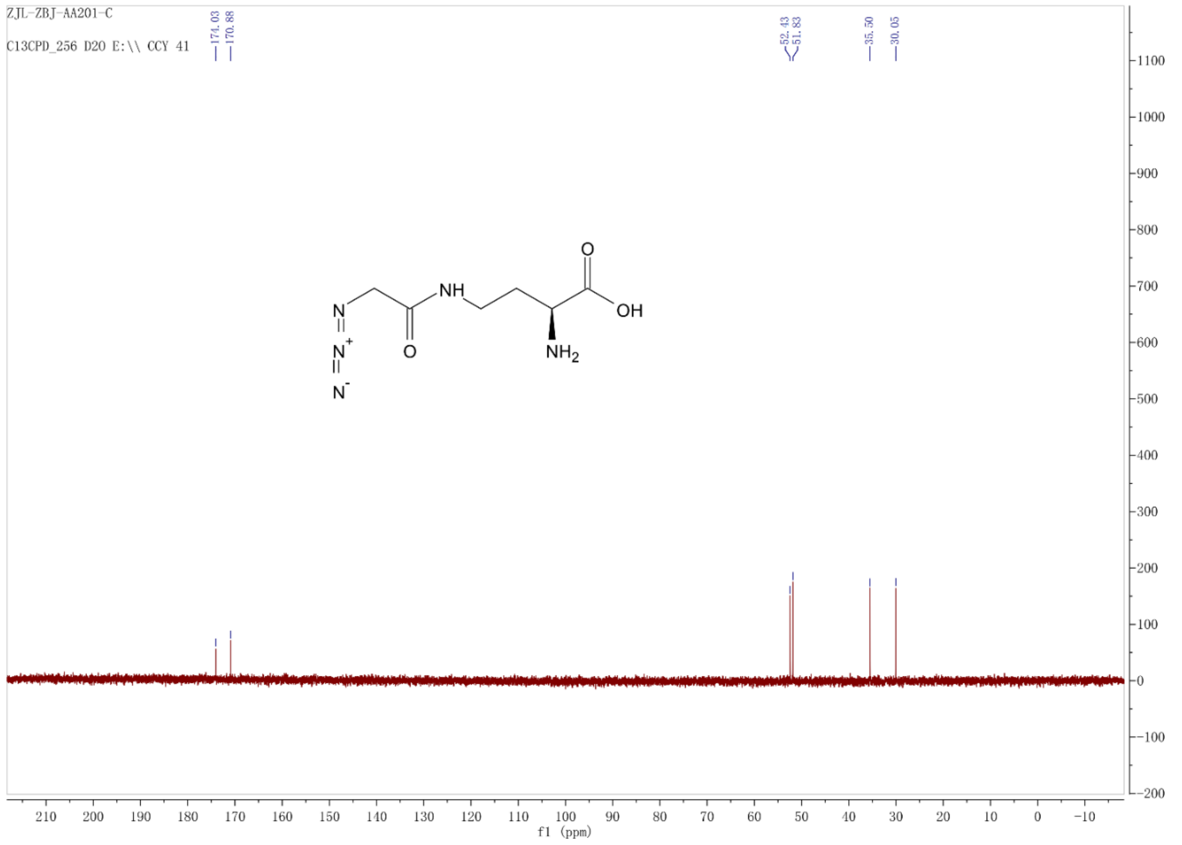

ZJL-ZBJ-AA201-C
C13CPD_256 D2O E:\\ CCY 41
174.03
170.88
52.43
51.83
35.50
30.05
O
NH
OH
N
N+
N-
O
NH2
f1 (ppm)

(c)

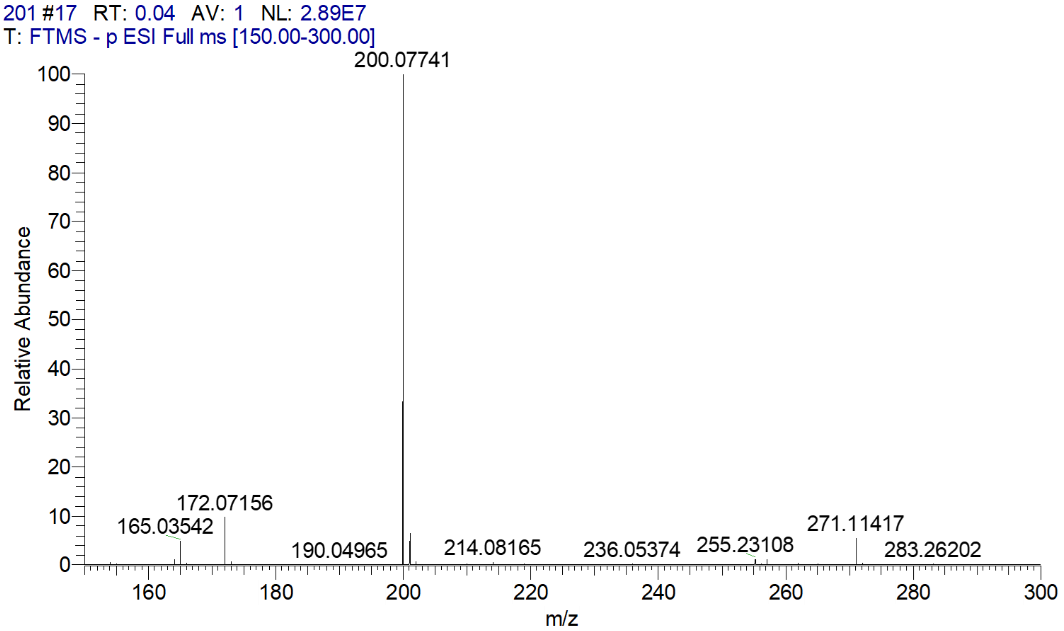

Figure S2. Structural characterization of AA201. (a) ^{1}H-NMR of AA201. (b) ^{13}C-NMR ofAA201. (c) High-resolution mass spectrum of AA201.

(a)

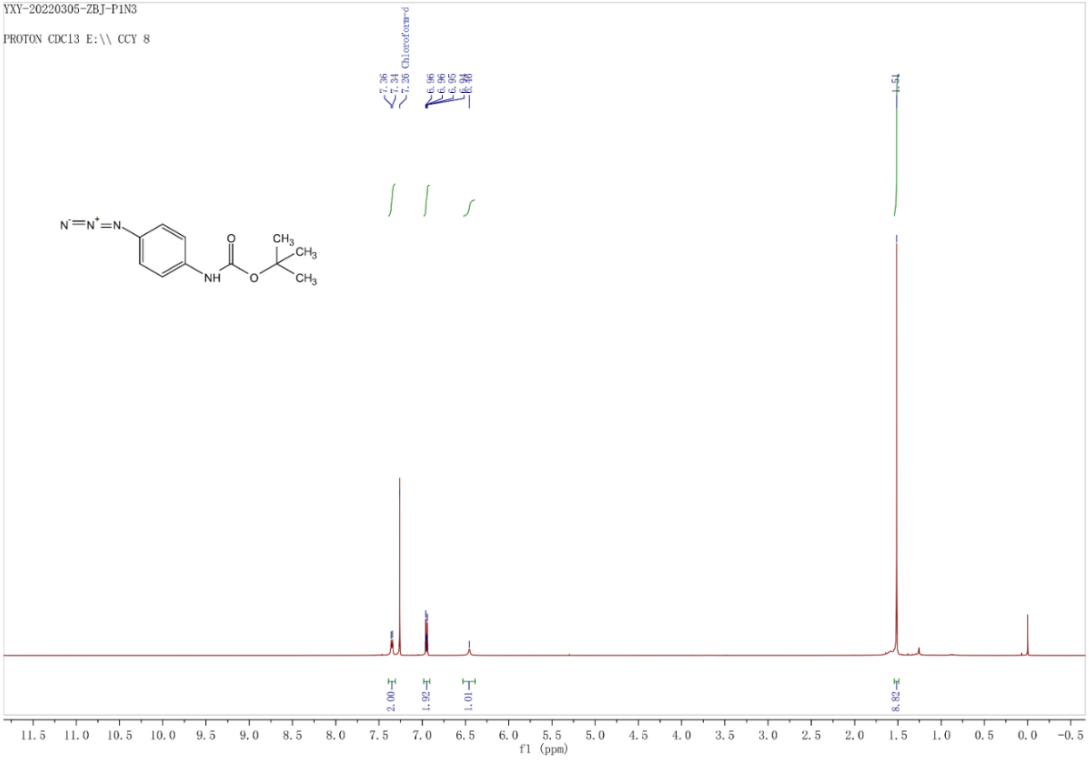

(b)

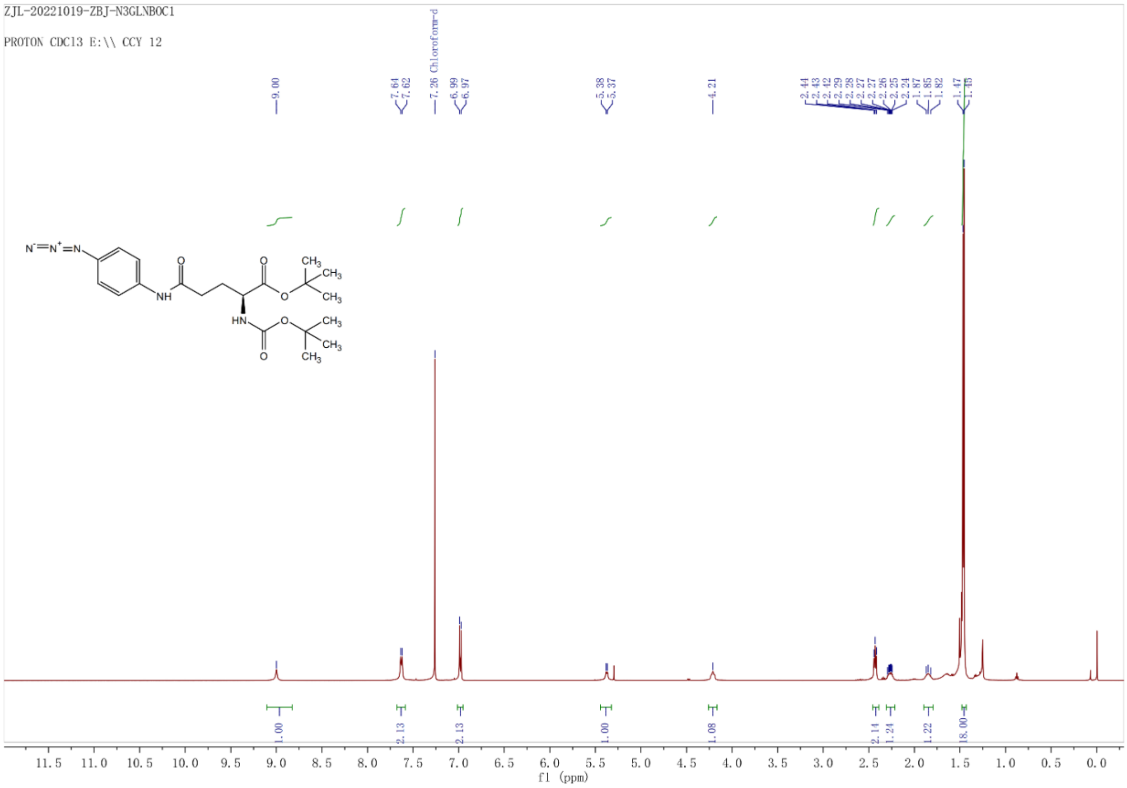

(c)

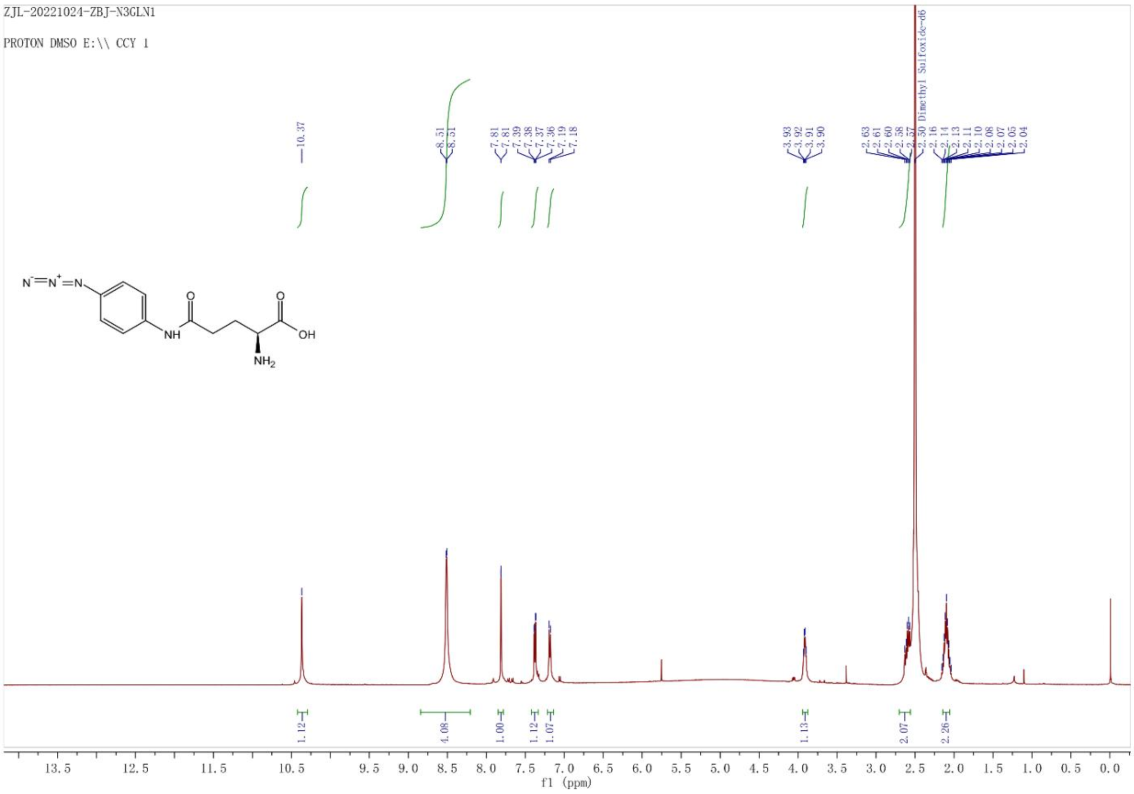

Figure S3. ^{1}H-NMR of (a) compound 234, (b) compound 419 and (c) AA263.

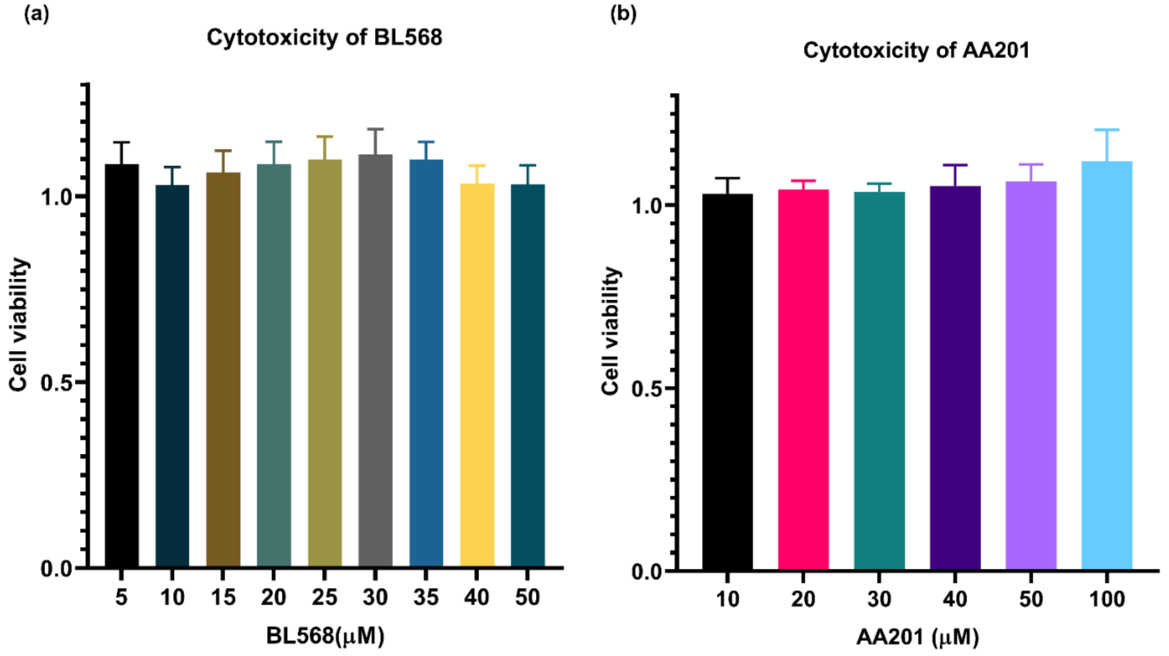

Figure S4. Cellular viability at different concentrations of (a) BL568 and (b) AA201. *n*=6.

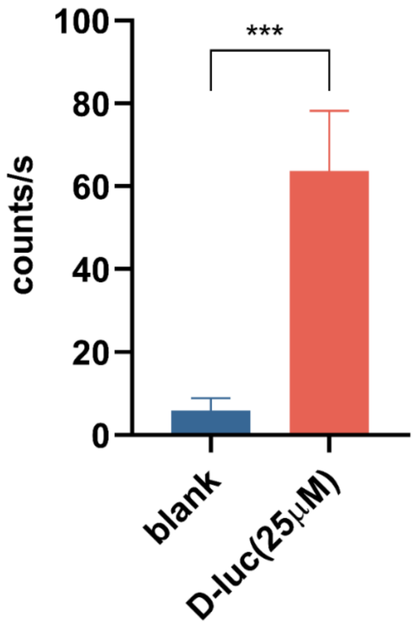

Figure S5. Verification of HCT116-luc cell line construction. *n*=6. *** *P* <0.001.

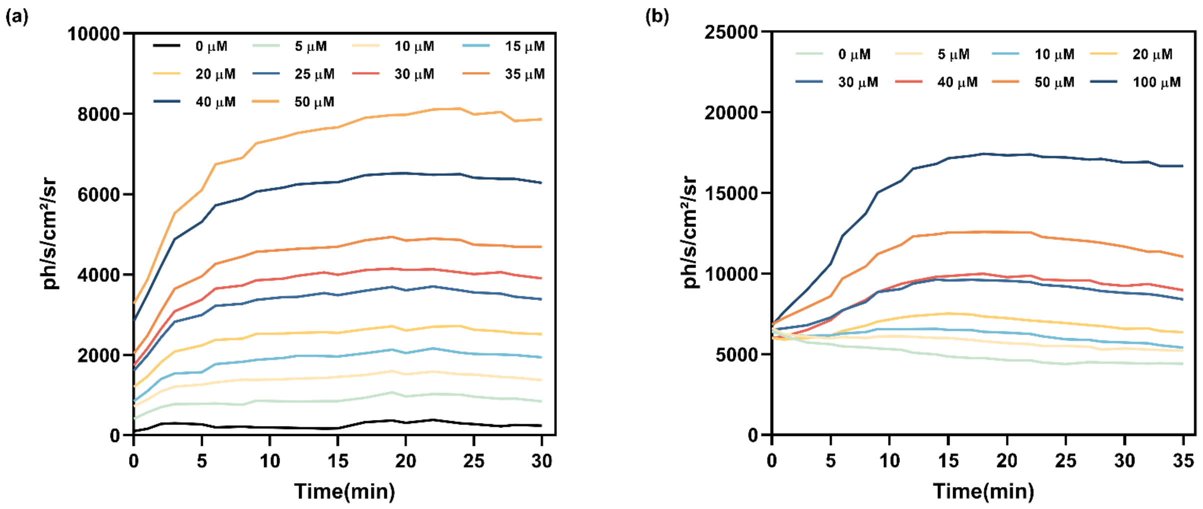

Figure S6. (a) BL intensity time course plot at different concentration of BL568. (b) BL intensity time course plot at different concentration of AA201. n=6.

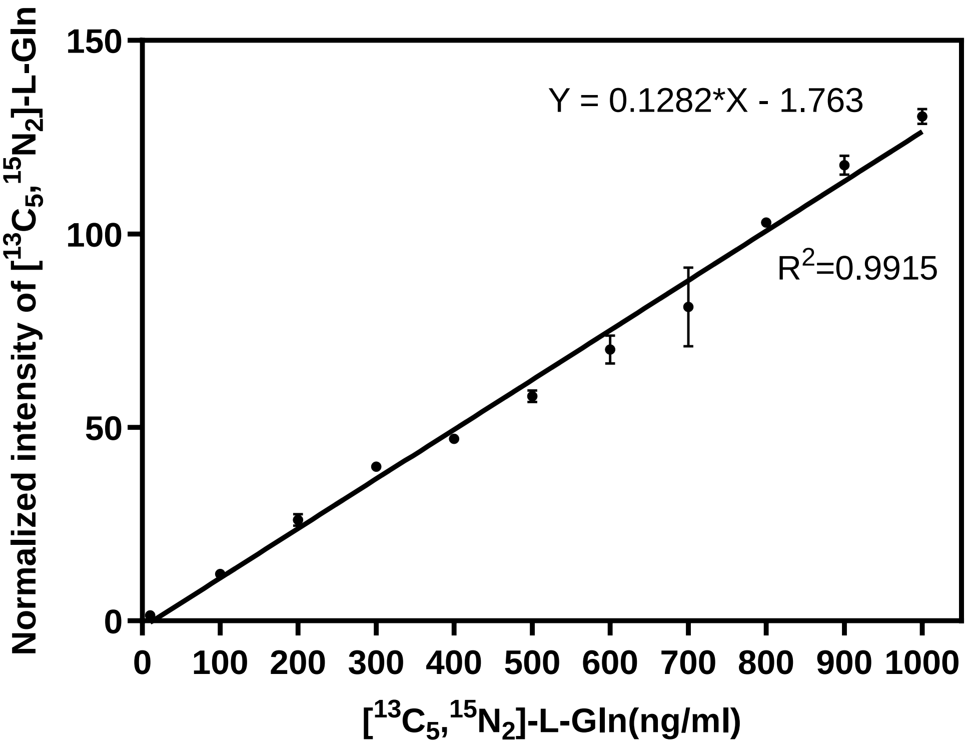

Figure S7. The standard curve of UPLC-QQQ-MS method to detect [$^{13}C_5$, $^{15}N_2$]-L-Gln.

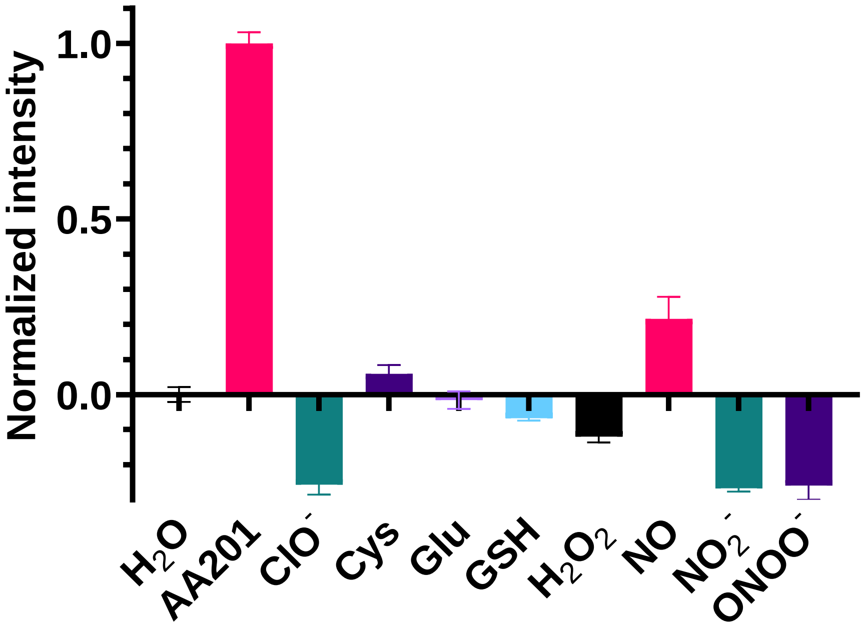

Figure S8. The selectivity of BL568 toward biorelevant interfering analytes (50μM, $ONOO^-$:1 μM, Cys: 1mM, GSH: 1mM, AA201: 500 μM). λex=310 nm, λem=530 nm.

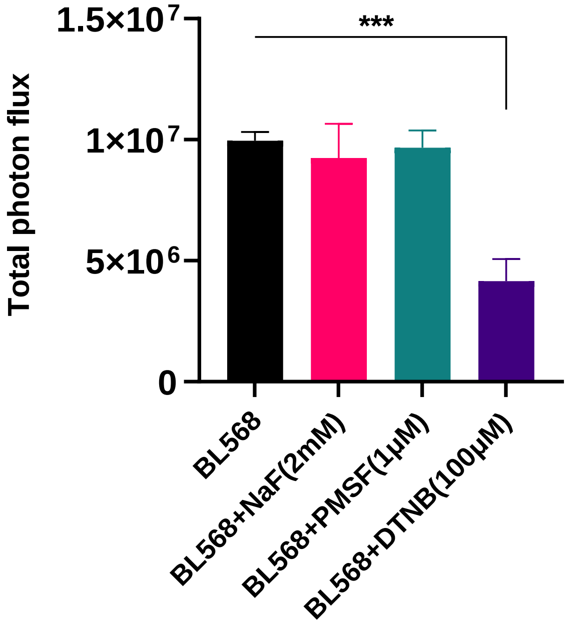

Figure S9. The effect of esterase inhibitors on the background signal of BL568. *** P <0.001

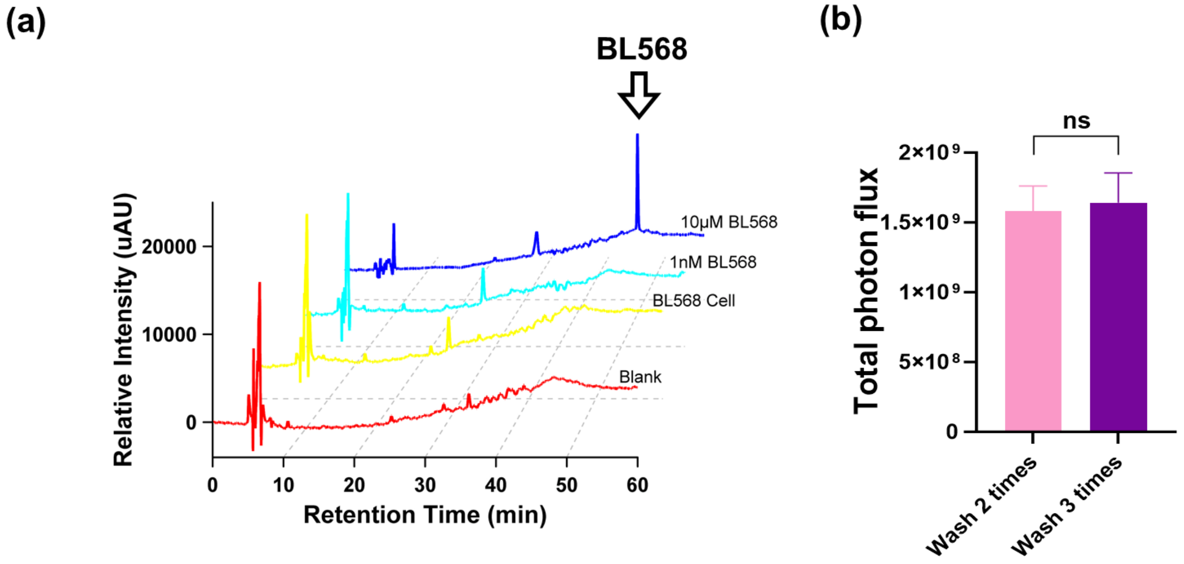

Figure S10. (a) HPLC chromatograms of extracellular residual BL568; (b)The total photon flux of different washing times on BLGLN. ns $P > 0.05$.

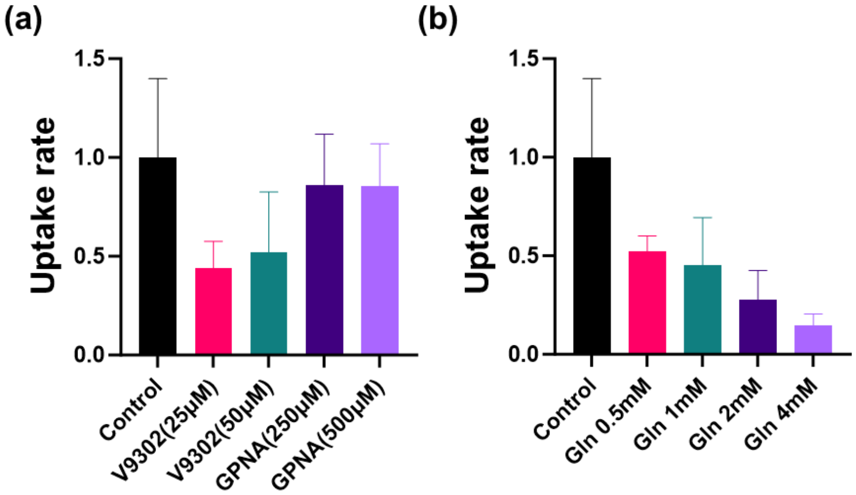

Figure S11. The uptake rate detected by BLGLN. (a) Evaluation of the effect of ASCT2 inhibitors on uptake rate. (b) Evaluation of the effect of glutamine in different concentrations on uptake rate.

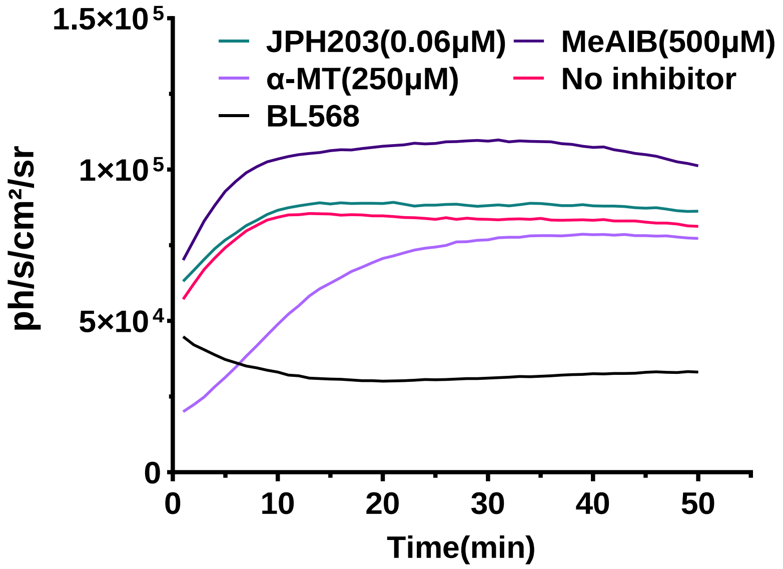

Figure S12. BL intensity time course in cells pre-treated with JPH203 (inhibitor of LAT1), MeAIB (inhibitor of SNATs) and α-MT (inhibitor of $ATB^{0,+}$).

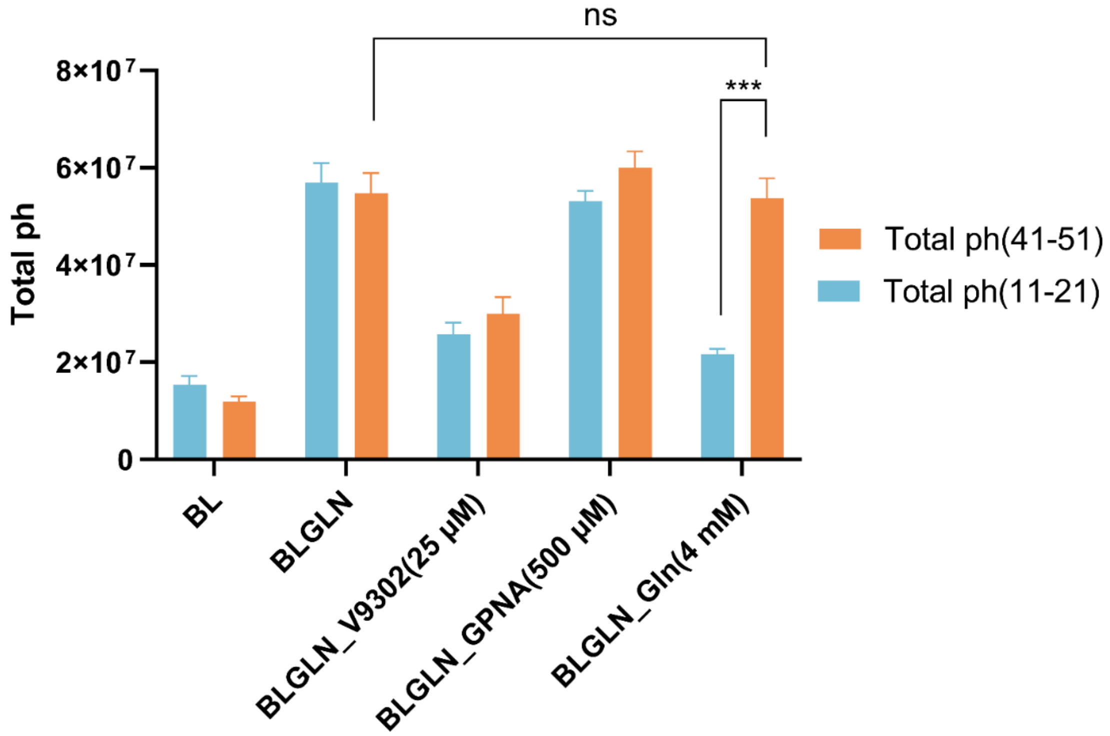

Figure S13. Total ph of 11 to 21 min and 41 to 51min. n=6. ns P >0.05, *** P < 0.001.
